# Coagulation proteases modulate nucleic acid uptake and cGAS-STING-IFN induction in the tumor microenvironment

**DOI:** 10.1172/jci.insight.190311

**Published:** 2025-07-22

**Authors:** Petra Wilgenbus, Jennifer Pott, Sven Pagel, Claudius Witzler, Jennifer Royce, Federico Marini, Sabine Reyda, Thati Madhusudhan, Thomas Kindler, Anne Hausen, Matthias M. Gaida, Hartmut Weiler, Wolfram Ruf, Claudine Graf

**Affiliations:** 1 Center for Thrombosis and Hemostasis, Johannes Gutenberg University Medical Center, Mainz, Germany.; 2 Department of Immunology and Microbiology, Scripps Research, La Jolla, California, USA.; 3 Institute of Medical Biostatistics, Epidemiology and Informatics (IMBEI),; 4 Research Center for Immunotherapy (FZI),; 5 Center for Translational Vascular Biology (CTVB), and; 6 University Cancer Center and 3rd Medical Department, Johannes Gutenberg University Medical Center, Mainz, Germany.; 7 German Cancer Consortium (DKTK), partner site Frankfurt/Mainz, a partnership between DKFZ and University Medical Center, Mainz, Germany.; 8 TRON, Translational Oncology at the University Medical Center, and; 9 Department of Pathology, Johannes Gutenberg University Medical Center, Mainz, Germany.; 10 Versiti Blood Research Institute and Medical College of Wisconsin, Department of Physiology, Milwaukee, Wisconsin, USA.

**Keywords:** Immunology, Oncology, Vascular biology, Cancer immunotherapy, Coagulation, Platelets

## Abstract

Malignancies increase the risk for thrombosis and metastasis dependent on complex interactions of innate immune cells, platelets, and the coagulation system. Immunosuppressive functions of platelets and macrophage-derived coagulation factors in the tumor microenvironment (TME) drive tumor growth. Here, we show that patients with malignancies and tumor-bearing mice have increased levels of coagulation factor (F) X–expressing circulating monocytes engaged in platelet aggregate formation. This interaction and resulting thrombin generation on platelets interferes with monocyte differentiation and antigen uptake of antigen-presenting cells (APCs). Myeloid cell–specific deletion of FX or abrogated FXa signaling via protease activated receptor 2 (PAR2) averts the suppressive activity of platelets on tumor cell debris uptake and promotes the immune stimulatory activity of APCs in the TME. Myeloid cell FXa-PAR2 signaling deficiency specifically enhances activation of the cGAS-STING-IFN-I pathway with a resulting expansion of antigen experienced progenitor exhausted CD8^+^ T cells. Pharmacological blockade of FXa with direct oral anticoagulants expands T cell priming–competent immune cells in the TME and synergizes with the reactivation of exhausted CD8^+^ T cells by immune checkpoint inhibitors for improved antitumor responses. These data provide mechanistic insights into the emerging clinical evidence demonstrating the translational potential of FXa inhibition to synergize with immunotherapy.

## Abstract 2

Targetable FXa-PAR2 signaling promoting monocyte-platelet aggregate formation subverts tumor DNA stimulation of the cGAS-STING-IFN pathway required for T cell priming and efficient checkpoint inhibitor therapy.

## Graphical abstract

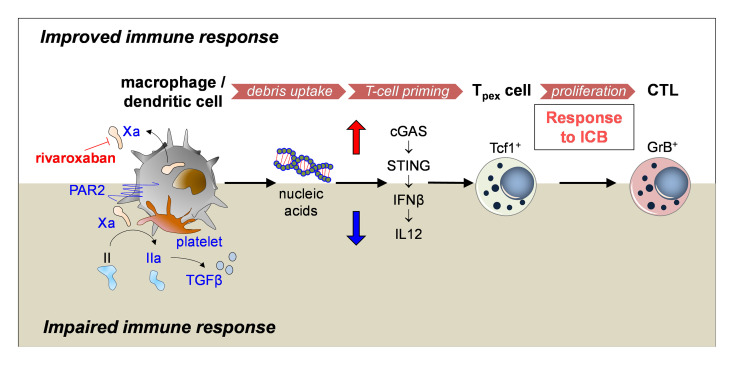

## Introduction

Malignancies remain a major cause of age-related mortality and are frequently associated with thrombosis due to a hyperactivated coagulation system. While obstruction of large peripheral veins and vessels in lungs is the major clinical manifestation of cancer associated thrombosis (CAT), microthrombi within tumors can impair tissue penetrance of drugs and thereby efficacy of systemic anticancer therapies (1). In addition, certain anticancer treatments can cause CAT and thereby even promote metastasis (2–4). Therefore, patients at high risk or with already established CAT are treated with anticoagulants, including direct oral anticoagulants (DOACs). The contribution of immune checkpoint blockade (ICB) to the development of CAT is still under debate (4–8). However, clinical data implicate a role for platelets and coagulation proteases in ICB efficacy, because patients who develop mild thrombocytopenia or receive DOACs during ICB show improved overall or progression-free survival (9–11). Tumor progression involves various microenvironments with distinct interactions affecting the immune cell landscape and coagulation with implications for responses to a broad range of cancer therapies (12–14). The hemostatic system, which is an integral component of innate immunity and tissue repair, is pivotal for the pathological processes in malignancy. For example, under physiological conditions, thrombin contributes to wound healing by generating fibrin but also by driving monocyte differentiation into the macrophage lineage (15) while impairing the differentiation into immunostimulatory functional dendritic cells (DCs) (16). Furthermore, the interaction of platelets with monocytes has been reported to propagate monocyte differentiation into macrophages (17) and to induce antiinflammatory and tolerogenic phenotypes (17–19). These findings demonstrate important local immunomodulatory functions of the hemostatic system in malignancies beyond the main clinical manifestations of a hypercoagulable state leading to CAT.

Indeed, tumors have been coined ‘wounds that do not heal’ (20, 21) due to visible local coagulation hyperactivation that not only deposits fibrin, but also alters local protease-dependent immunomodulatory signaling involving monocytes, tumor associated macrophages (TAMs) (22), and platelets (23). Platelets are a major source of TGFβ (24) which can be released by thrombin-mediated cleavage of Glycoprotein A Repetitions Predominant (GARP), an anchor protein for TGFβ, and of the latency associated protein (LAP) of TGFβ (23). Platelet-derived TGFβ contributes to the immunosuppressive character of the TME by inducing fibrosis and preventing immune cell infiltration (23). Furthermore, TGFβ impairs the efficacy of immunotherapies with ICB or bispecific antibodies (23, 25). Treatment response to both can be improved by therapy with the thrombin inhibitor dabigatran (23, 25).

TAMs typically form the most prevalent immune cell population within the TME and are highly heterogenous. TAMs are major drivers of intratumoral coagulation activation and the major extrahepatic cellular synthesis source for coagulation proteases. TAMs express key components of coagulation signaling complexes, including FVII, FX, and their cognate receptors, tissue factor (TF), endothelial protein C receptor (EPCR), and protease activated receptor (PAR) 2 (26). As we have previously demonstrated, macrophage autonomous FXa-PAR2 signaling favors immunosuppression in the TME, tumor growth, and spontaneous metastasis to the lungs. Blocking FXa with the tissue penetrant direct oral FXa inhibitor rivaroxaban reverses the immune-suppressive effects of macrophages and increases the accumulation of cytotoxic CD8^+^ T cells (CTL) in the TME (26).

Moreover, recent studies support the translational relevance of these findings (10, 23, 26, 27). DOACs not only showed synergistic effects with immune checkpoint blockade (ICB) in mice (23, 26), but FXa inhibitors also improved progression free survival (PFS) in patients with metastatic melanoma who were treated with ICB in a single center retrospective study (10). These beneficial effects on PFS and tumor progression were not seen with any other class of anticoagulants. Beneficial synergistic effects of FXa inhibition are not limited to immunotherapy but were also observed with enzalutamide in castration-resistant prostate cancer in preclinical models (27). Furthermore, FXa inhibitors reduce the prometastatic effects of chemotherapy mediated by upregulated *F10* expression by macrophages in destined metastatic niches (2).

In this study, we systematically probe the interplay of monocyte/macrophage–derived FXa and platelet bound (pro)thrombin in suppressing antitumor responses by antigen presenting cells (APCs). These experiments uncover crucial roles of platelet-dependent thrombin generation, myeloid cell–derived FXa and FXa-PAR2 signaling in monocyte-platelet aggregate formation, propagating monocyte differentiation into functionally impaired APCs. Those compromised APCs show reduced antigen uptake, cGAS-STING-IFN induction, and, in consequence, less capacity in fostering the expansion of antigen-experienced progenitor exhausted CD8^+^ T cells. Loss of FXa expression or FXa-PAR2 signaling by myeloid cells averts this immunosuppressive effect and provides a mechanism by which the pharmacological inhibition of FXa by DOACs alters function of APCs in the TME and thus allows the expansion of progenitor exhausted CD8^+^ T cells to synergize with clinically relevant ICB.

## Results

### FXa-PAR2 signaling modulates nucleic acid uptake and cGAS-STING-IFN-I induction in vivo.

**Figure 1:**
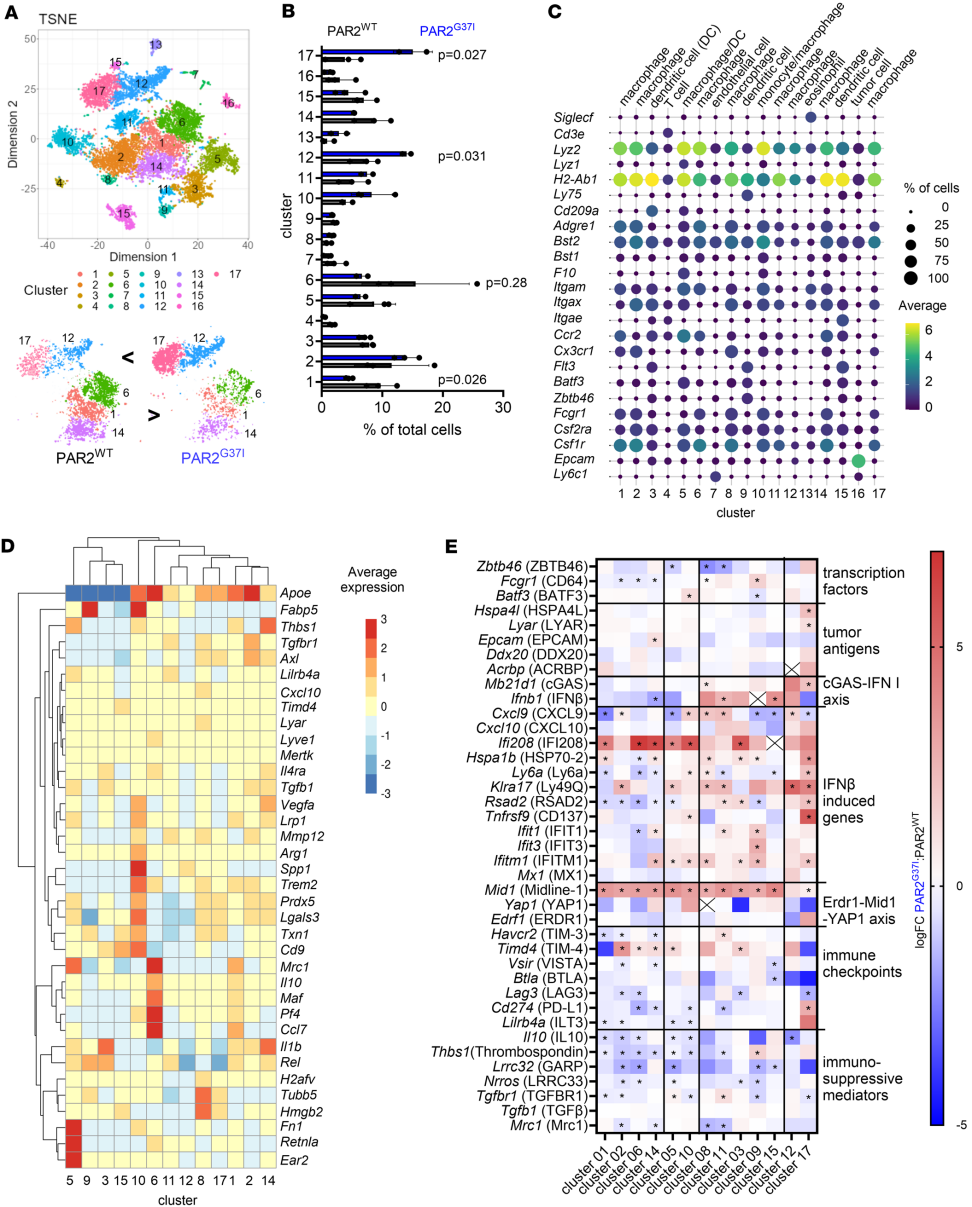
FXa-PAR2 signaling modulates macrophage phenotypes in the TME. (**A**) t-SNE embedding of single-cell sequenced CD11c selected cells from PyMT-PAR2^WT^ and PyMT-PAR2^G37I^ tumors colored by cluster assignment; merged data sets of *n =* 3 per genotype. (**B**) Relative abundance of cells in the different clusters from PyMT-PAR2^WT^ versus PyMT-PAR2^G37I^ TME; *n =* 3, mean ±SD, 2-sided *t* test. (**C**) Dot plot of average expression (color coded) of marker genes for macrophages, DCs, T cells, endothelial cells, and eosinophils and percentage (coded by dot size) of cells expressing these markers in the same clusters. (**D**) Heatmap showing average expression of marker genes for clusters classified as macrophages and DCs. (**E**) Heatmap showing (log fold change; logFC) differential expression between genotypes of transcripts encoding for immune checkpoints, immune mediators, transcription factors (TFs) relevant for DC differentiation, and tumor antigens; *n =* 3, *P_adj.loc_ < 0.05

Myeloid cell autonomous FXa-PAR2 signaling mediates immunosuppression in several spontaneous and transplanted tumor models (26). To specifically investigate FXa-PAR2 signaling–dependent alterations of tumor infiltrating macrophage and DC phenotypes, we compared single cell RNA sequencing (scRNA-seq) profiles of CD11c-selected cells from the TME of the spontaneous breast cancer model PyMT, developing in FXa signaling–resistant PAR2^G37I^ and signaling-competent PAR2^WT^ mice (26), later referred to as PyMT-PAR2^G37I^ or PyMT-PAR2^WT^. Seventeen distinct cell clusters were identified by t-distributed Stochastic Neighbor Embedding (tSNE) (Figure 1A). The TME of PyMT-PAR2^WT^ mice had a higher cell abundance in clusters 1 and 6, whereas the TME of PyMT-PAR2^G37I^ mice was enriched in cluster 12 and 17 cells (Figure 1B). The different clusters were classified based on cell type defining markers (28–30) as macrophages (clusters 1, 2, 6, 8, 11, 12, 14, 17), DCs (cluster 3, 9, 15), monocyte/macrophage (cluster 10), macrophage/DC (cluster 5), or copurifying T cells (cluster 4), endothelial cells (cluster 7), eosinophils (cluster 13), and tumor cells (cluster 16) (Figure 1C). The top 5 defining marker genes of innate immune cell clusters are shown in Supplemental Figure 1A (supplemental material available online with this article; https://doi.org/10.1172/jci.insight.190311DS1). High expression of *F10* mRNA and *F7* mRNA, encoding for the FX activating protease FVII*,* was detected in clusters 5 and 10 (Figure 1C and Supplemental Figure 1B).

Besides phenotypic macrophage markers (Figure 1, C and D), cluster 10 cells were enriched in transcripts involved in oxidative stress and lipid metabolism (*Fabp5*, *Txn1*, *Lgals3*, *Prdx5*), thus resembling lipid-associated (31) or TREM2^hi^ macrophages (32). Cluster 5 cells showed transcripts (Figure 1, C and D) characteristic for early-stage monocytes differentiating into macrophages (33) and already expressed the TME macrophage marker *Mrc1*. However, cluster 5 cells also expressed *Cd209a* (DC SIGN), a marker of immature and monocyte-derived DCs (moDCs) in peripheral tissues (34). Importantly, cluster 5 and 10 cells, expressing *F10,* from the TME of PyMT-PAR2^G37I^ mice had significantly lower expression of *Lilrb4a* (Lilrb4, ILT3), *Il10*, and of genes involved in TGFβ release and signaling, such as *Thbs1* (thrombospondin 1), *Nrros* (Lrrc33), *Lrrc32* (GARP), and *Tgfbr1*. Expression of these genes cause immune suppression, because ILT3 together with fibronectin (*Fn1*) and the TGFβ receptor Lrrc33 function as checkpoints suppressing myeloid cells (35). IL10 and TGFβ enhance expression of thrombospondin 1, which, in turn, promotes IL10 and TGFβ release and is a binding partner for CD41/CD61 on platelets (36–38).

Cluster 1 and 6 cells, which were expanded in PyMT-PAR2^WT^mice, had transcript profiles (Figure 1D) resembling resident-like and infiltrating macrophage subsets, also found in cardiovascular diseases and premalignant lesions (31, 32). Cluster 6 cells showed the highest expression of *Pf4*. Pf4-expressing macrophages were recently described to promote tumor growth via T_H1_-T_reg_ polarization (39). Furthermore, cluster 14 cells, which were also somewhat more prominent in PyMT-PAR2^WT^ mice, expressed *Vegfa*, *Thbs1*, *Tgfb1*, *Tgfbr1,* and *Lilrb4a,* implicated in neoangiogenesis, metastasis, and immunosuppression (40, 41). In addition to downregulated immunosuppressive transcripts (*Mrc1, Il10*), several macrophage subsets in the TME of PyMT-PAR2^G37I^ mice also showed lower expression of immune checkpoints (*Havcr2*, *Vsir*, *Lag3*, *Cd274*), but upregulated *Timd4* (T cell immunoglobulin and mucin domain containing 4, TIM4) (Figure 1E). TIM4 is important for capture and engulfment of tumor cell–associated antigens and responses to αPD1 treatment (42).

Cluster 17 cells expressed transcripts (Figure 1D) that were consistent with an oxidative stress and cycling phenotype, but also autophagy and DNA sensing (33). Remarkably, we found higher transcript levels of PyMT tumor antigens (43, 44) (*Hspa4l*, *Lyar*, *Ddx20*, *Acrbp*) in these cells of PyMT-PAR2^G37I^ relative to PyMT-PAR2^WT^ mice (Figure 1E), suggesting increased uptake of tumor cell debris, which contains various nucleic acid species. Among these are RNA:DNA hybrids that can be detected during RNA-seq. Similar to dsDNA, RNA:DNA hybrids bind to the cyclic GMP-AMP synthase (cGAS), triggering its activation and leading to the production of cGAMP and subsequent activation of the stimulator of interferon genes (STING) (45). Stimulation of the cGAS-STING pathway in APCs mediates antitumor immunity through type-I interferon (IFN-I) production — namely IFNβ — promoting CD8^+^ T cell priming (46, 47). Increased *Mb21d1* (cGAS) expression in cluster 17 cells from the PyMT-PAR2^G37I^ TME compared with WT indicated activation of this pathway. We also found higher expression of IFN-induced genes, such as *Klra17* (48), *Ly6a* (49), *Rsad2,* and *Tnfrsf9* (50, 51) in cluster 17 cells from mutant mice (Figure 1E). Higher transcript levels of IFN-induced genes were not limited to cells in cluster 17, but were also detected in other macrophage and DC clusters of PyMT-PAR2^G37I^ mice. The IFN-inducible genes *Ifi208* (52) and *Mid1* (53) were not only upregulated on a per cell basis, but the abundance of cells expressing *Ifif208* and *Mid1* was also increased in PyMT-PAR2^G37I^ compared with PyMT-PAR2^WT^ mice (Figure 1E and Supplemental Figure 1C).

Expression of transcription factors (*Zbtb46*, *Fcgr1*, *Batf3*) in cluster 9 and 10 cells relevant for moDC and classical DC differentiation (54) suggested a shift to moDCs, which express *Itgae*, in the TME of PyMT-PAR2^G37I^ mice (Figure 1, C and E). In addition, upregulation of *Ifnb1* (cluster 3, 8, 11, 12, 15) and *Cxcl9* (cluster 8, 10, 11, 12), both relevant for IL-12 induction in DCs, indicated improved T cell priming in FXa-PAR2 signaling deficient mice.

Interestingly, impaired FXa-PAR2 signaling also affected the phenotype of contaminating *Cd44/Epcam*^+^ tumor cells (cluster 16). In PyMT-PAR2^G37I^ mice, these had lower transcript levels implicated in epithelial-to-mesenchymal transition (EMT), metastasis, proliferation, and stem cell signature (Supplemental Figure 1D) found in PyMT cancer cells (55). The indicated reduced metastatic potential was in line with macrophage FX–deficient PyMT mice displaying less spontaneous lung metastases (26).

### Myeloid cell FX deficiency and abrogated FXa-PAR2 signaling cause similar quantitative shifts in myeloid subpopulations in the TME.

**Figure 2:**
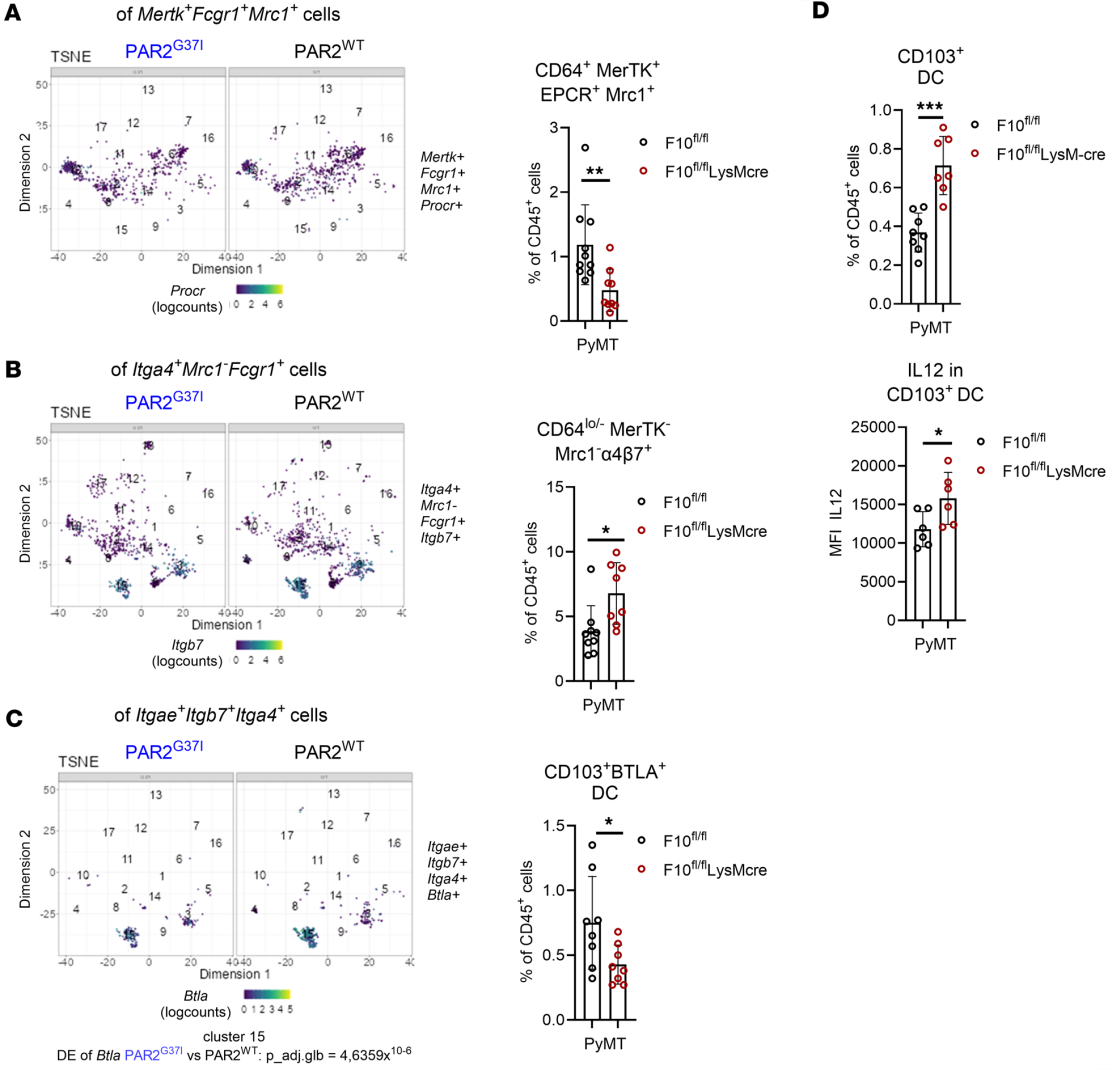
Myeloid cell FX deficiency and abrogated FXa-PAR2 signaling cause similar quantitative shifts in myeloid subpopulations in the TME. (**A**–**C**) Coexpression analysis as determined by scRNA-seq in CD11c-selected cells from PyMT-PAR2^WT^ and PyMT-PAR2^G37I^ tumors (*n =* 3; see also Supplemental Figure 1D) and by flow cytometric quantification of TAMs and DCs from the TME of PyMT-F10^fl/fl^LysMcre and F10^fl/fl^ littermate control mice; *n =* 8–10, mean ± SD, 2-sided unpaired *t* test. (**A**) *Mertk*, *Fcgr1*, *Mrc1* and *Procr* mRNA or CD64, MerTK, EPCR, Mrc1 protein coexpression. (**B**) Cells with the mRNA marker profile *Itga4^+^Mrc1^–^Fcgr^+^Itgb7^+^* or the protein surface profile CD64^lo/–^MerTK^–^Mrc1^–^α4β7^+^. (**C**) Cells with the mRNA marker profile *Itgae^+^Itgb7^+^Itga4^+^Btla^+^* or the protein surface profile CD11c^+^CD103^+^Btla^+^. (**D**) Flow cytometry quantification of total and IL12 expressing CD103^+^ DCs in the TME of PyMT-F10^fl/fl^LysMcre or PyMT-F10^fl/fl^ littermate control mice; *n =* 6-8, mean ± SD, 2-sided unpaired *t* test. *P < 0.05; **P < 0.01; ***P < 0.001.

Myeloid cell FX deficiency phenocopies the reduced tumor growth seen in FXa-PAR2 signaling–defective mice (26). We next analyzed the TME of cohoused PyMT-F10^fl/fl^LysMcre mice and PyMT-F10^fl/fl^ littermate controls by flow cytometry for phenotypic changes in macrophage and DC subsets. For the identification of macrophage subpopulations, we used CD64 (*Fcgr1*), MerTK, EPCR (*Procr*), and Mrc1 (Supplemental Figure 2A and Figure 2A), characterizing cluster 1 and 6 cells with reduced abundance in PyMT-PAR2^G37I^ versus PyMT-PAR2^WT^ mice in the scRNA-seq data set. Although the flow cytometry gating strategy had lower resolution to dissect macrophage heterogeneity than the scRNA-seq (Supplemental Figure 2B), the TME of PyMT-F10^fl/fl^LysMcre mice showed reduced CD64/MerTK/EPCR/Mrc1^+^ macrophages (Figure 2A) which also encompassed *Mertk/Fcgr1* expressing cluster 2 and 14 cells and the *F10* expressing cluster 5 and 10 cells in the scRNA-seq data of PyMT-PAR2^G37I^ and PyMT-PAR2^WT^ mice. Conversely, CD64^lo/–^MerTK^–^Mrc1^–^α4β7^+^ macrophage/DC subsets matching cells from cluster 3, 9, 10, 12, 15, and 17 (Figure 2B and Supplemental Figure 2B) were increased in the TME of PyMT-F10^fl/fl^LysMcre mice (Figure 2B). Moreover, the frequency of CD103^+^ DC-expressing BTLA, an immune checkpoint relevant for tolerance induction, was reduced in the TME of PyMT-F10^fl/fl^LysMcre mice, fitting to lower expression levels of *Btla* in cluster 3 and 15 cells of PyMT-PAR2^G37I^ versus PyMT-PAR2^WT^ mice (Figure 2C). Thus, flow cytometry of myeloid cell FX-deficient mice indicated a shift in macrophage populations concordant with FXa-PAR2 signaling–deficient mice. However, while the scRNA-seq data did not reveal a quantitative difference in *Itgae*-expressing DCs in PyMT-PAR2^G37I^ mice compared with PyMT-PAR2^WT^ mice, PyMT-F10^fl/fl^LysMcre mice had higher frequencies of CD103^+^ DCs that also showed higher IL12 production (Figure 2D).

### Myeloid cell–expressed FXa is crucial for nucleic acid uptake by antigen presenting cells.

**Figure 3:**
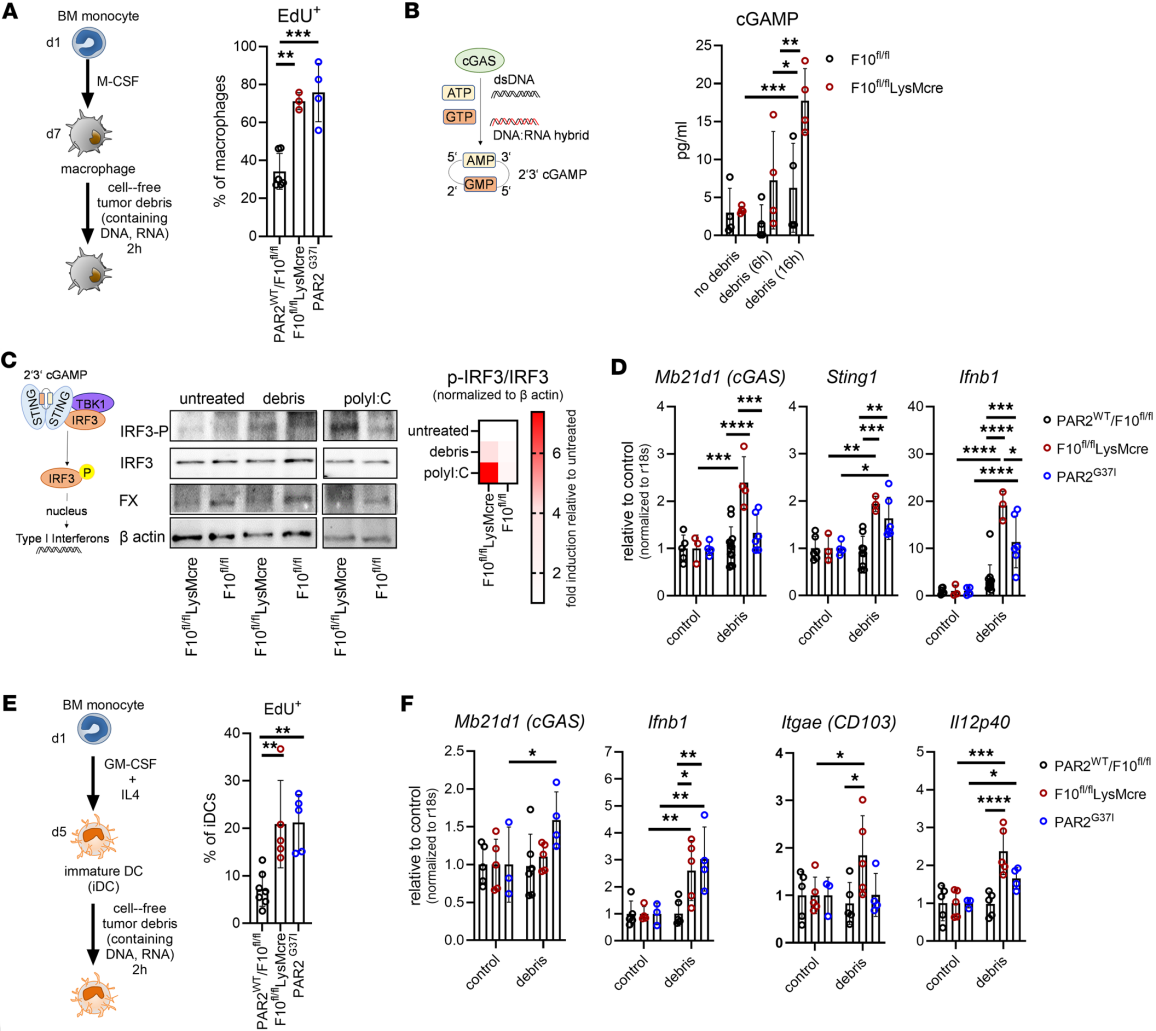
Myeloid cell derived FXa is crucial for nucleic acid uptake by antigen presenting cells and cGAS-STING-IFN-I induction. (**A**) EdU labeled cell free tumor debris uptake for 2 hours in BM monocyte derived macrophages from PAR2^WT^/F10^fl/fl^, PAR2^G37I^, or F10^fl/fl^LysMcre mice determined by flow cytometry; *n =* 3–6, 1-way ANOVA with Dunnett’s multiple comparison test. (**B**) Intracellular cGAMP produced by macrophages from *F10^fl/fl^* or F10^fl/fl^LysMcre mice after tumor debris loading for 6 or 16 hours versus untreated controls; *n =* 4, 2-way ANOVA with Sidak’s multiple comparison test. (**C**) Western blot showing phospho-IRF3 (IRF3-P), IRF3, and FX protein expression by macrophages from F10^fl/fl^ or F10^fl/fl^LysMcre mice with loading control β actin. Cells were either untreated or loaded with tumor debris or polyI:C (25 μg/mL) as positive controls for phosphor-IRF3 for 90 minutes. Lanes were run on the same gel but were noncontiguous. (**D**) *Mb21d1, Sting1,* and *Ifnb1* mRNA expression normalized to r18s by PAR2^WT^/F10^fl/fl^, PAR2^G37I^ or F10^fl/fl^LysMcre derived macrophages with and without tumor cell debris loading for 2 hours; *n =* 3–12, 2-way ANOVA with Sidak’s multiple comparison test. (**E**) Tumor debris uptake determined by flow cytometry of iDCs from PAR2^WT^, PAR2^G37I^, F10^fl/fl^, or F10^fl/fl^LysMcre; *n =* 5–7, 1-way ANOVA with Dunnett’s multiple comparison test. (**F**) *Mb21d1, Sting1, Ifnb1, Itgae and Il12p40* mRNA expression normalized to r18s by PAR2^G37I^, F10^fl/fl^ vs F10^fl/fl^LysMcre derived iDCs with and without tumor debris loading for 2 hours; *n =* 3–6, 2-way ANOVA with Sidak’s multiple comparison test. *P < 0.05 **P < 0.01; ***P < 0.001; ****P < 0.0001.

We next asked whether the suggested improved uptake of tumor cell debris and activation of the cGAS-STING-IFN-I pathway in vivo resulted from the loss of cell intrinsic FXa-PAR2 signaling. Therefore, we differentiated BM monocytes from F10^fl/fl^LysMcre, PAR2^G37I^ and strain matched control WT mice with macrophage colony stimulating factor (M-CSF) into macrophages, which upregulated relevant coagulant factors during differentiation (Supplemental Figure 3A). We used EdU labeled tumor cells to isolate cell-free debris containing EdU^+^ DNA, RNA, and RNA:DNA hybrids and proteins, and quantified EdU^+^ DNA uptake efficiency by a Click-IT EdU reaction in flow cytometry (56). Macrophages lacking FXa-PAR2 signaling or FX expression showed an improved uptake of cell-free tumor debris (Figure 3A). This led to increased cGAS-dependent cGAMP production (Figure 3B) and IRF3 phosphorylation (Figure 3C) as shown as an exemplary for F10^fl/fl^LysMcre vs F10^fl/fl^ mice. In line with the presented in vivo data for FXa-PAR2 cleavage–resistant mice (Figure 1E), macrophages from myeloid cell FX-deficient and FXa-PAR2 signaling–impaired mice showed upregulated mRNA expression of *Mb21d1*, *Sting1*, and *Ifnb1*, indicating increased activation of the cGAS-STING-IFN-I pathway (Figure 3D).

Induction of the cGAS-STING-IFN-I pathway stimulates antitumor immune responses in vivo (46, 47). We therefore differentiated bone marrow (BM) monocytes into immature DCs (iDCs), which also expressed relevant coagulation factors during differentiation (Supplemental Figure 3B). Cell-free tumor debris uptake by PAR2^G37I^ and F10^fl/fl^LysMcre iDC was markedly enhanced relative to controls (Figure 3E). In line with the increased tumor debris uptake, the cGAS-STING-IFN pathway resulting in increased *Il12p40* mRNA expression was also induced in iDCs lacking FX expression or FXa-PAR2 signaling (Figure 3F). Furthermore, iDCs of F10^fl/fl^LysMcre but not of PAR2^G37I^ mice showed an upregulated *Itgae* mRNA expression (Figure 3F). This observation was in line with higher frequencies of CD103^+^ DCs in the TME of tumor-bearing mice in F10^fl/fl^LysMcre compared with littermate controls (Figure 2D) whereas the abundance of *Itgae*-expressing DCs in clusters 3 and 9 in the TME of PAR2^G37I^ mice did not differ from WT mice (Figure 1B).

### Myeloid cell–derived FXa recruits immune suppressive platelets.

**Figure 4:**
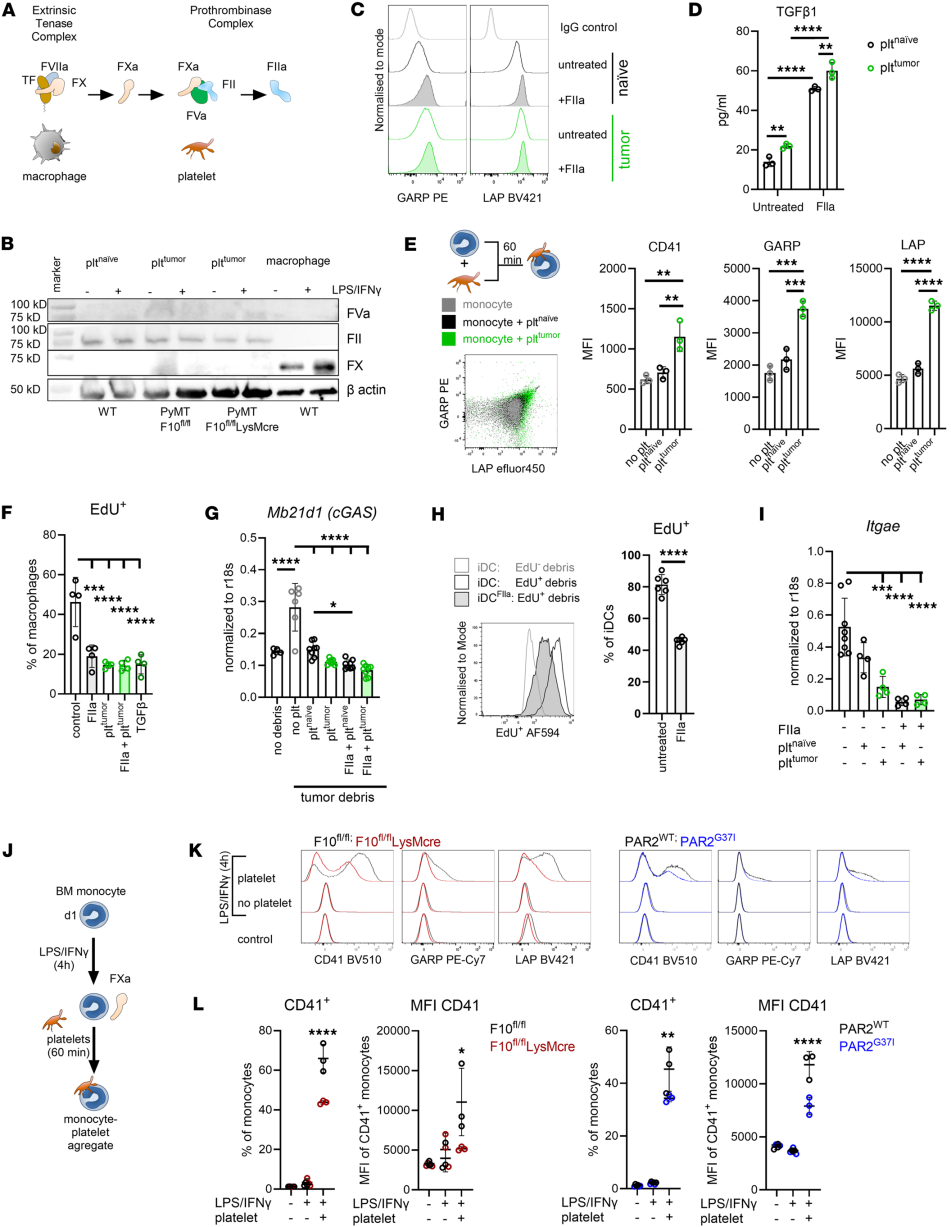
Myeloid cell–derived FXa recruits immune suppressive platelets. (**A**) Schematic overview of coagulation initiation by macrophage-expressed FX by the TF-FVIIa complex and prothrombin (FII) to thrombin (FIIa) activation by platelet localized FVa and FXa. (**B**) Western blot showing a representative example of FX, FII, and FVa protein expression by platelets from tumor free (plt^naive^) and tumor-bearing (plt^tumor^) PyMT-F10^fl/fl^ or PyMT-F10^fl/fl^LysMcre mice and by in vitro–generated macrophages from a WT mouse normalized to β actin. Cells were stimulated with LPS/IFNγ overnight to assess the effects of an inflammatory environment on protein expression. (**C**) GARP and LAP on platelets from tumor free (naive) or tumor bearing (tumor) mice with or without thrombin (FIIa) activation shown as histograms. (**D**) Quantification of released active TGFβ1 from the experiment shown in **C**; *n =* 3, mean ± SD, 2-way ANOVA with Sidak’s multiple comparison test. (**E**) GARP and LAP/TGFβ on the monocyte surface after 1 hour of incubation with platelets from tumor free (naive) or tumor bearing (tumor) mice. Shown is a representative dot plot and the quantification of CD41, GARP, and LAP surface levels on monocytes; *n =* 3, mean ± SD, 1-way ANOVA with Dunnett’s multiple comparison test. (**F**) Uptake of EdU labeled cell free tumor debris by BM monocyte derived WT macrophages after 2 hours of incubation. Macrophages were differentiated in the absence (control) or presence of thrombin (FIIa), platelets from tumor free (plt^naïve^) or tumor bearing (plt^tumor^) mice, or TGFβ; *n =* 2–4, mean ± SD, 1-way ANOVA with Dunnett’s multiple comparison test. (**G**) Quantification of *Mb21d1* mRNA expression normalized to r18s in WT macrophages, differentiated as indicated, after exposure to cell free tumor debris for 6 hours. *n =* 4–8, mean ± SD, 1-way ANOVA with Dunnett’s multiple comparison test. (**H**) Flow cytometry analysis of cell free EdU labeled tumor debris uptake after 2 hours by iDCs differentiated with or without thrombin (FIIa) *n =* 6, mean ± SD, 2-sided unpaired *t* test. (**I**) Quantification of *Itgae* mRNA expression by iDCs normalized to r18s in WT iDCs differentiated as indicated; *n =* 4–8, mean ± SD, 1-way ANOVA with Dunnett’s multiple comparison test. (**J**) Experimental set up for monocyte-platelet aggregate formation between BM monocytes from F10^fl/fl^ and F10^fl^LyscMcre or PAR2^WT^ and PAR2^G37I^ mice and WT platelets after 60 minutes of coculture. Monocytes were pre-incubated for 4 hours with LPS/IFNγ to induce FX expression. (**K** and **L**) Shown are representative histograms for surface levels of CD41, GARP, and LAP (**K**) and quantification of CD41^+^ monocytes and surface levels of CD41 (**L**) on these cells; *n =* 3, mean ± SD, 2-way ANOVA with Sidak’s multiple comparison test. *P < 0.05; **P < 0.01; ***P < 0.001; ****P < 0.0001.

Macrophage FX is typically activated by coagulation factor VIIa (FVIIa) in complex with TF. FXa, in turn, binds to activated factor V (FVa) as part of the prothrombinase complex, which subsequently catalyzes the conversion of prothrombin (FII) to thrombin (FIIa) (Figure 4A). TF, FVII, and FX were expressed by monocyte-derived macrophages and DCs (Supplemental Figure 3, A and B), whereas prothrombin was not (Supplemental Figure 4A). As previously described, protein expression of FX by macrophages can be induced by inflammatory LPS/IFNγ priming (26). In contrast, platelets from naive or tumor-bearing mice contained FVa and prothrombin but no FX, irrespective of inflammatory priming (Figure 4B). Although tumor cells are a known source of prothrombin (57), our tumor models showed only very low prothrombin (*F2*) mRNA expression and no detectable prothrombin protein in cell lysates (Supplemental Figure 4, A and B). We hypothesized that macrophage-derived FXa could trigger thrombin generation on the platelet surface expressing FVa. Indeed, reactions of M-CSF differentiated and LPS/IFNγ-primed macrophages stimulated thrombin generation only when platelets were present (Supplemental Figure 4C). In addition, thrombin generation was markedly reduced in macrophages derived from F10^fl/fl^LysMcre but not FXa-PAR2 signaling–deficient PAR2^G37I^ mice (Supplemental Figure 4C). Thrombin is a potent platelet activator and cleaves GARP on the platelet surface, thereby liberating TGFβ (23). Accordingly, the interaction of macrophages with platelets triggering thrombin generation resulted in the release of active TGFβ into the supernatant (Supplemental Figure 4D).

Platelet-derived TGFβ has been demonstrated to be the dominant source of functional TGFβ systemically and in the TME and to be bound to the platelet TGFβ docking receptor GARP (24). Circulating platelets from tumor-bearing mice expressed higher surface levels of GARP and LAP/TGFβ (Figure 4C), and isolated platelets spontaneously released higher levels of active TGFβ than platelets from naive mice, which were significantly enhanced upon exposure to thrombin (Figure 4D). In addition, thrombin stimulation increased platelet GARP and LAP/TGFβ surface levels, especially on platelets from naive mice (Figure 4C). Furthermore, addition of platelets isolated from tumor-bearing mice to BM monocytes for 60 minutes resulted in aggregate formation with much higher GARP and LAP/TGFβ expression than observed upon addition of naive platelets (Figure 4E). In line with prior studies (58), thrombin stimulation of naive platelets in vitro resulted in a dose- and time-dependent formation of monocyte aggregates detected by the platelet surface markers CD41, LAP, and GARP (Supplemental Figure 4E). Thus, thrombin stimulation of platelets or in vivo priming of platelets in tumor-bearing mice increased monocyte aggregate formation. Aggregate formation was markedly reduced when monocytes and platelets were cocultured in the presence of the FXa inhibitor rivaroxaban or the thrombin inhibitor hirudin (Supplemental Figure 5A).

We next asked whether aggregate formation with platelets could alter the phenotypes of in vitro–differentiated DCs and macrophages. Macrophage differentiation in the presence of platelets, thrombin, or active TGFβ significantly reduced the uptake of EdU^+^ tumor debris compared with control macrophages (Figure 4F). Reduced nucleic acid uptake also correlated with diminished induction of *Mb21d1* mRNA encoding cGAS (Figure 4G). Differentiation of iDCs in the presence of thrombin showed a similar impairment of tumor debris uptake (Figure 4H). Furthermore, iDCs differentiated in the presence of platelets from tumor-bearing, but not from naive, mice showed reduced *Itgae* (*Cd103*) mRNA expression (Figure 4I). Thus, platelets, and potentially platelet-mediated thrombin generation, interfere with crucial initial steps in tumor-antigen uptake and priming of antitumor responses.

Activated platelets also form aggregates with circulating monocytes. We next asked whether the association of platelets with monocytes was dependent on monocyte synthesized FX. We isolated monocytes from the BM of F10^fl/fl^, F10^fl/fl^LysMcre, PAR2^WT^, and PAR2^G37I^ mice and stimulated them with LPS/IFNγ for 4 hours to induce FX expression, followed by incubation with platelets for 60 minutes to allow for thrombin generation, platelet activation, and aggregate formation (Figure 4J). Cells were washed to stop further aggregate formation and analyzed by flow cytometry for CD41, GARP, and LAP accumulation on the monocyte surface. CD41, GARP, and LAP were only detectable upon addition of platelets (Figure 4K). FX-deficient monocytes formed fewer CD41^+^ monocyte-platelet aggregates and bound significantly less CD41. Remarkably, monocytes from FXa-PAR2 signaling–deficient PAR2^G37I^ mice also formed significantly reduced numbers of monocyte-platelet aggregates, indicating that PAR2 signaling primed monocytes for platelet interaction (Figure 4L). Similarly, the accumulation of tumor-induced platelet markers GARP and LAP were also reduced on monocytes from F10^fl/fl^LysMcre and PAR2^G37I^ mice (Figure 4K).

### FX regulates monocyte-platelet aggregate formation during tumor progression.

**Figure 5:**
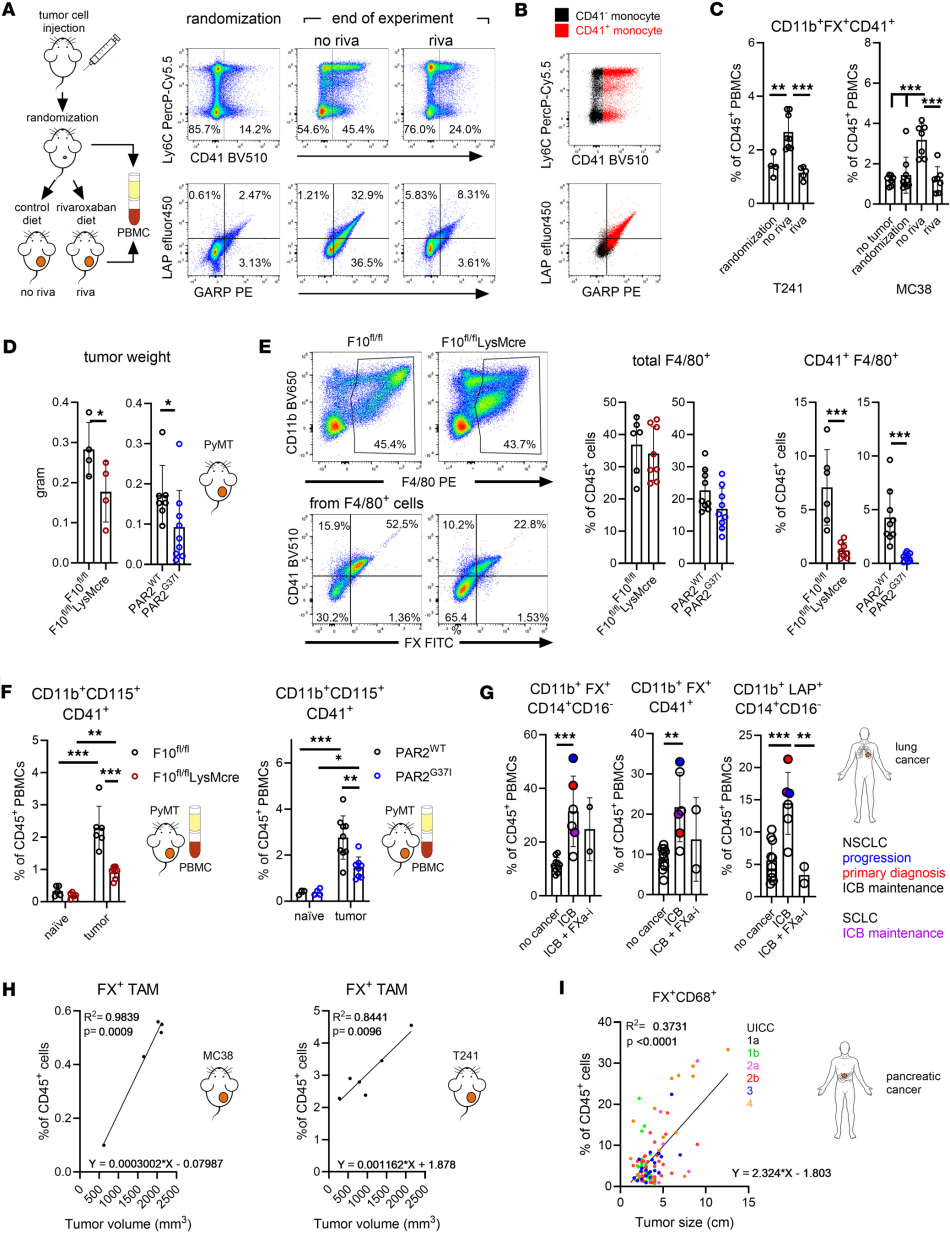
Myeloid cell–expressed FX regulates monocyte-platelet aggregate formation during tumor progression. (**A**–**C**) T241 or MC38 tumor cells were injected in WT mice. Once tumors reached palpable size, mice were randomized into control or rivaroxaban treatment group. Blood was taken from different animals at randomization or at the end of the experiment. PBMCs were isolated and analyzed by flow cytometry for monocyte-platelet aggregates. (**A**) Representative dot plots of LAP/TGFβ versus GARP and Ly6C versus CD41 surface expression on PB monocytes after injection of T241 cells at randomization (*n =* 4) and at the end of the experiment of control (*n =* 8) or rivaroxaban (*n =* 5) treated mice. (**B**) Determination of LAP and GARP surface expression on CD41^–^ or CD41^+^ monocytes from the control treated group as shown in **A**. (**C**) Quantification of FX^+^ monocyte:platelet aggregates dependent on tumor stage and treatment with rivaroxaban; T241: *n =* 4-8, MC38: *n =* 7–9, mean ± SD, 1-way ANOVA with Dunnett’s multiple comparison test. (**D**–**F**) F10^fl/fl^, F10^fl/fl^LysMcre and PAR2^WT^, PAR2^G37I^ mice were s.c. injected with PyMT tumor cells. (**D**) Tumor weights at sacrifice; *n =* 4–9, mean ± SD, 2-sided unpaired *t* test. (**E**) Flow cytometric quantification of total and CD41^+^ macrophages in the TME; *n =* 6–9, mean ± SD, 2-sided unpaired *t* test. Shown are representative dot plots of macrophages from the TME of F10^fl/fl^ and F10^fl/fl^LysMcre mice. (**F**) Flow cytometric quantification of CD41^+^ monocytes in the peripheral blood of naive or tumor-bearing mice at the end of the experiment.; F10^fl/fl^, F10^f/fll^LysMcre*:* *n =* 5–8, PAR2^WT^, PAR2^G37I^: *n =* 3–9, 2-way ANOVA with Sidak’s multiple comparison test. (**G**) Flow cytometric quantification of FX^+^, CD41^+^ or LAP/TGFβ^+^ PB monocytes in patients with lung cancer at various stages of tumor progression before and under ICB treatment relative to healthy controls. Different treatment stages are marked with different colors; *n =* 2–10. (**H**) Simple linear regression analysis of FX^+^ macrophages versus total tumor volume (TTV) in the TME of WT mice after injection of T241 (*n =* 6) or MC38 (*n =* 5) tumor cells. (**I**) Simple linear regression analysis of FX^+^ macrophages versus tumor size in the TME of pancreatic ductal adenocarcinoma (PDAC) patients (*n =* 106). Each dot is color coded according to the patient’s UICC (Union for International Cancer Control) classification. *P < 0.05 **P < 0.01; ***P < 0.001; ****P < 0.0001.

To investigate the relevance of the platelet aggregate supporting role of monocyte-derived FX and FXa-PAR2 signaling in tumor progression, we measured the abundance of FX-expressing monocytes in the peripheral blood of tumor-bearing WT mice. The frequency of FX^+^ monocytes increased with tumor burden (Supplemental Figure 5B). In addition, FX^+^ monocytes stained all positive for CD41 (Supplemental Figure 5B), in line with the in vitro data demonstrating a role for monocyte-expressed FX in monocyte-platelet aggregate formation (Figure 4K). We also detected higher surface expression of LAP/GARP on peripheral blood monocytes during tumor progression (Figure 5A) and only CD41^+^ monocytes were LAP^+^GARP^+^ (Figure 5B). In line with prior studies in patients with cardiovascular disease (59), treatment of mice with rivaroxaban reduced the frequency of CD41^+^ monocyte-platelet aggregates that also stained positive for LAP and GARP (Figure 5, A and C).

We next studied monocyte-platelet aggregates in mice with subcutaneously implanted PyMT breast cancer cells. In line with spontaneously developing breast cancer (26), PyMT tumors grew slower in mice lacking myeloid FX expression or FXa-PAR2 signaling (Figure 5D). Although the frequencies of all macrophages in the TME did not differ between F10^fl/fl^ and F10^fl/fl^LysMcre or PAR2^WT^ and PAR2^G37I^ mice, the frequencies of CD41^+^ macrophages were markedly reduced upon abrogated myeloid FX expression or FXa-PAR2 signaling (Figure 5E). In addition, the frequency of CD41^+^ monocytes in the peripheral blood of tumor-bearing mice was significantly lower in myeloid FX or FXa-PAR2 signaling–deficient mice compared with the respective control mice (Figure 5F).

Increased circulating monocyte-platelet aggregates were also detected in human patients with lung cancer. In this explorative study, the patients with untreated disease at primary diagnosis or with progressive disease showed the highest abundance of FX^+^ monocytes, as well as monocytes forming aggregates with platelets, compared with healthy controls (Figure 5G). Patients receiving direct oral FXa inhibitors had a lower abundance of monocyte-platelet aggregates that were also characterized by LAP surface expression. Thus, we could verify our preclinical results of increased frequencies of FX-expressing peripheral blood monocytes and FXa-regulated monocyte-platelet aggregate formation in patients with cancer. In addition, the abundance of FX^+^ TAM in the TME of mice with different tumor models (Figure 5H) as well as in the TME of patients with pancreatic cancer (Figure 5I) increased with tumor size, indicating that expansion of FX^+^ TAM is a potential marker for tumor progression.

### Progenitor exhausted CD8^+^ T cells are expanded in FXa-PAR2 signaling–deficient mice.

**Figure 6:**
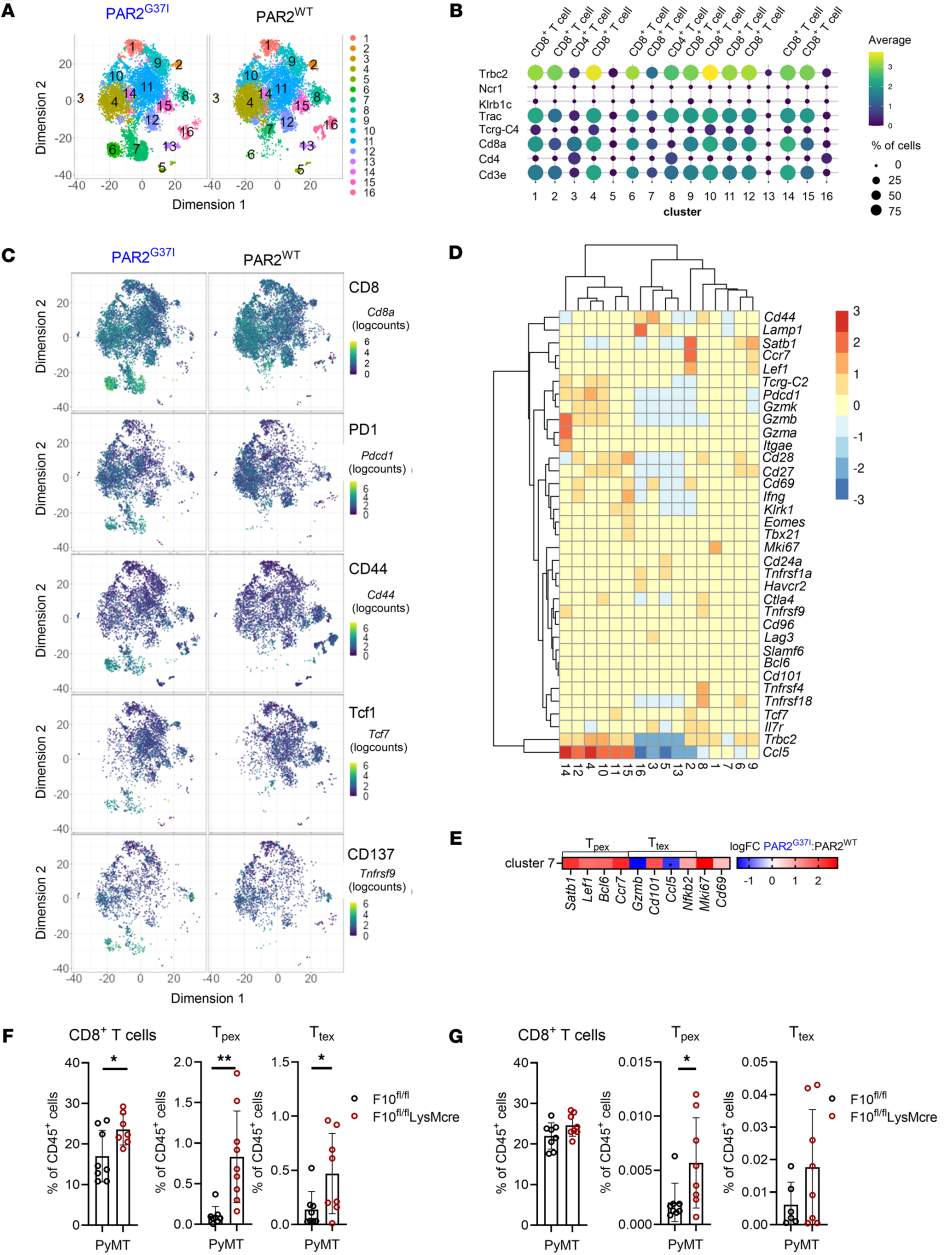
Progenitor exhausted CD8^+^ T cells are expanded in FXa-PAR2 signaling deficient mice. (**A**) tSNE embedding of single cell sequenced CD4/CD8 selected cells from PyMT-PAR2^WT^ and PyMT-PAR2^G37I^ tumors colored by cluster assignment; merged data sets of *n =* 3 per genotype. (**B**) Dot plot of average expression (color coded) of T cell markers (*Trbc2, Trac, Tcrg-C4, Cd8a, Cd4, Cd3e*) and NK cell markers (*Ncr1, Klrb1c*) and percentage (coded by dot size) of cells expressing these markers in the same clusters. (**C**) tSNE plots of cells expressing markers of progenitor and terminally exhausted CD8^+^ T cells (*Pdcd1*, *Cd44*, *Tcf7*), IFNAR2 (*Ifnar2*) and of *Tnfrsf9* (CD137), indicating recent antigen recognition and activation. (**D**) Heatmap showing average expression of genes marking progenitor or terminally exhausted CD8^+^ T cells in these clusters. (**E**) Differential transcript abundance of genes known to be predominantly expressed in progenitor or terminally exhausted CD8^+^ T cells in cluster 7 cells of PyMT-PAR2^WT^ and PyMT-PAR2^G37I^ tumors; *n* = 3; * *P*_loc.adj_ < 0.05. (**F** and **G**) Frequencies of total, progenitor (T_pex_: Tcf1^+^Tim3^–^PD1^+^CD44^+^) and terminally (T_tex_: Tcf1^–^Tim3^+^ PD1^+^CD44^+^) exhausted CD8^+^ T cells in the TME (**F**) and tumor draining lymph nodes (**G**) of PyMT-F10^fl/fl^LysMcre or PyMT-F10^fl/fl^ littermate control mice; *n =* 6–8, mean ± SD, 2-sided unpaired *t* test. *P < 0.05; **P < 0.01

cGAS-STING–induced IFN-I increases the expression of the transcription factor TCF1, and, in consequence, the activity of stem-like CD8^+^ T cells (60) that are capable of self renewal and differentiation into effector cells (61). To investigate the consequences of the observed enhanced cGAS-STING-IFN-I induction in APCs present in the TME of FXa-PAR2 signaling–deficient mice, we characterized the phenotypes of CD8^+^ T cells by generating scRNA-seq profiles of tumor-infiltrating lymphocytes from the TME of PyMT transgenic mice. PyMT-PAR2^G37I^ mice showed an overall similar abundance and phenotype of T cell subsets as PyMT-PAR2^WT^ mice, except for clusters 6 and 7, which were more prominent in PAR2^G37I^ mice and markedly reduced in PAR2^WT^ mice (Figure 6A).

Cells from these clusters expressed TCRβ chain (*Trbc2)* and *Cd8a* (Figure 6B) as well as markers defining progenitor and terminally exhausted T cells (Figure 6, C and D) (61). In line with the role of IFN-I in inducing TCF1 expression, these cells also expressed *Ifnar1* and *Ifnar2* required for IFN-I signaling. In addition, the expression of *Tnfrsf9* (CD137) indicated a recent antigen recognition (62). Evaluation of additional markers for exhausted T cell subsets (63) showed higher expression of *Satb1* and *Cd28* in cluster 6 cells compared with cluster 7, suggesting a progenitor phenotype (Figure 6D). Cluster 7 cells from PAR2^WT^ mice expressed higher amounts of *Ccl5,* consistent with terminally exhausted T cells (Figure 6E). In contrast, cluster 7 cells from PAR2^G37I^ mice had higher transcript levels related to progenitor status (*Satb1*, *Lef1*, *Bcl6*, *Ccr7*) (61) and lower expression of terminally differentiated cell markers (*Gzmb*, *Ccl5*) (61) relative to PAR2^WT^ mice (Figure 6E). These cells also showed higher transcript levels of *Tbx21*, *Tnf,* and *Tcf7* in PAR2^G37I^ mice and therefore fitted best to an intermediate exhausted phenotype (63). In a next step, we verified altered T cell phenotypes in the TME (Figure 6F) and tumor draining lymph nodes (Figure 6G) of PyMT-F10^fl/fl^LysMcre versus littermate control PyMT-F10^fl/fl^ mice. CD8^+^ T cell counts and frequencies of progenitor (T_pex_) and terminally (T_tex_) exhausted CD8^+^ T cells were also increased in the TME and tumor-draining lymph nodes of myeloid cell FX deficient mice.

**Figure 7:**
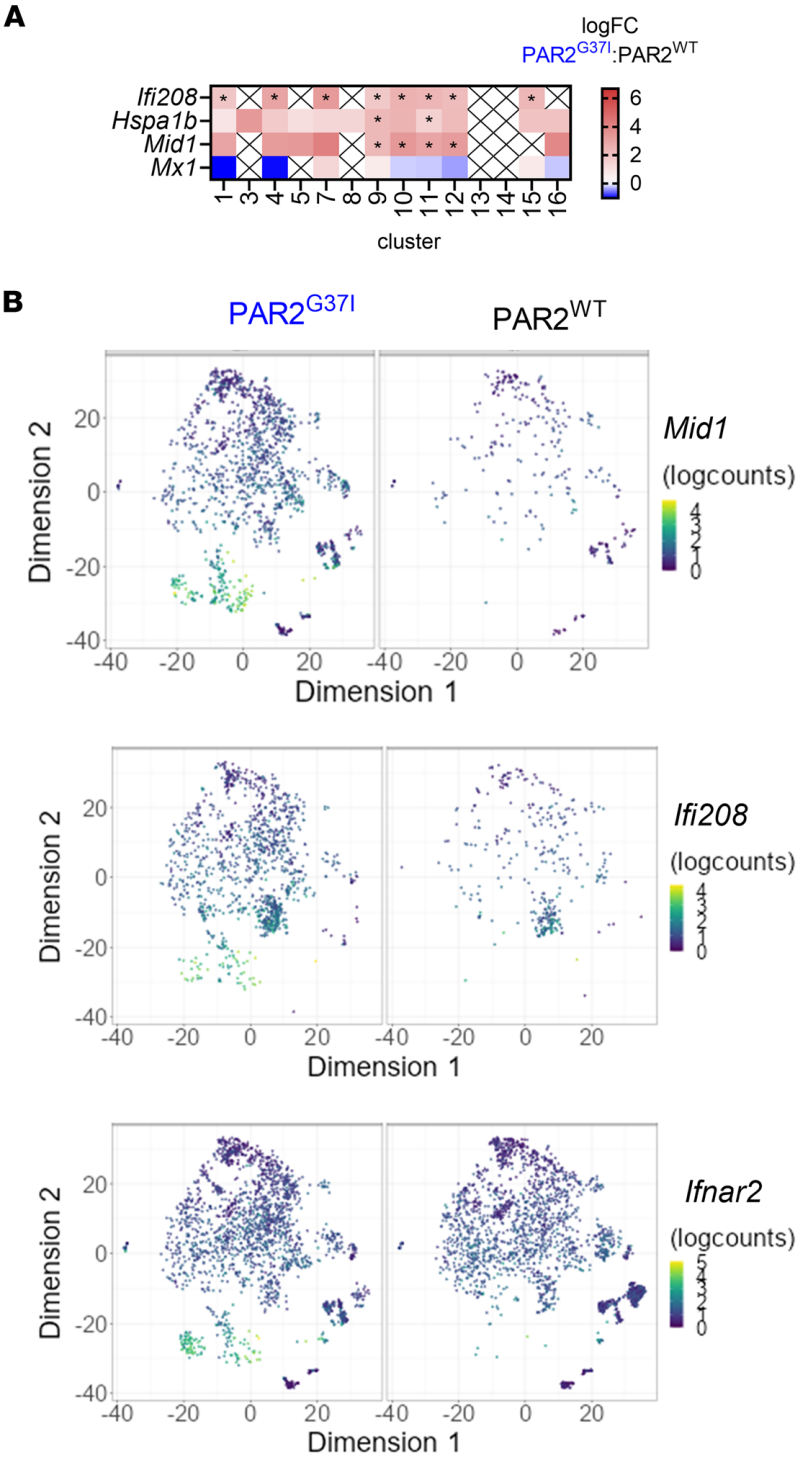
CD8^+^ T cells in FXa-PAR2 signaling–deficient mice show IFN-I induced activation pattern. (**A**) Differential transcript abundance of IFN inducible genes in T cells of the various clusters of PyMT-PAR2^WT^ and PyMT-PAR2^G37I^ tumors; *n* = 3; *P_adj_ < 0.05. (**B**) tSNE plots of cells expressing IFNAR2 (*Ifnar2*) and of IFN-induced genes *Ifi208* (IFI208) and *Mid1* (Midline 1) indicating IFN-I induced activation.

Activation of the cGAS-STING-IFN pathway in the APCs from the TME of PyMT-PAR2^G37I^ mice also caused higher transcript levels of IFN-induced genes in CD8^+^ T cells of PyMT-PAR2^G37I^ mice compared with PyMT-PAR2^WT^ mice (Figure 7A). Especially, *Ifi208* and *Mid1* were expressed by more T cells and at higher transcript levels per T cell in PyMT-PAR2^G37I^ compared with PyMT-PAR2^WT^ mice (Figure 7, A and B). It is known that Midline 1 controls polarization and migration of T cells as well as exocytosis of lytic granules (64, 65). Thus, loss of myeloid cell FXa-PAR2 signaling led to an expansion and activation of antigen-experienced T cells in the TME.

### FXa inhibition synergizes with immune checkpoint blockade to expand progenitor-exhausted CD8^+^ T cells.

**Figure 8:**
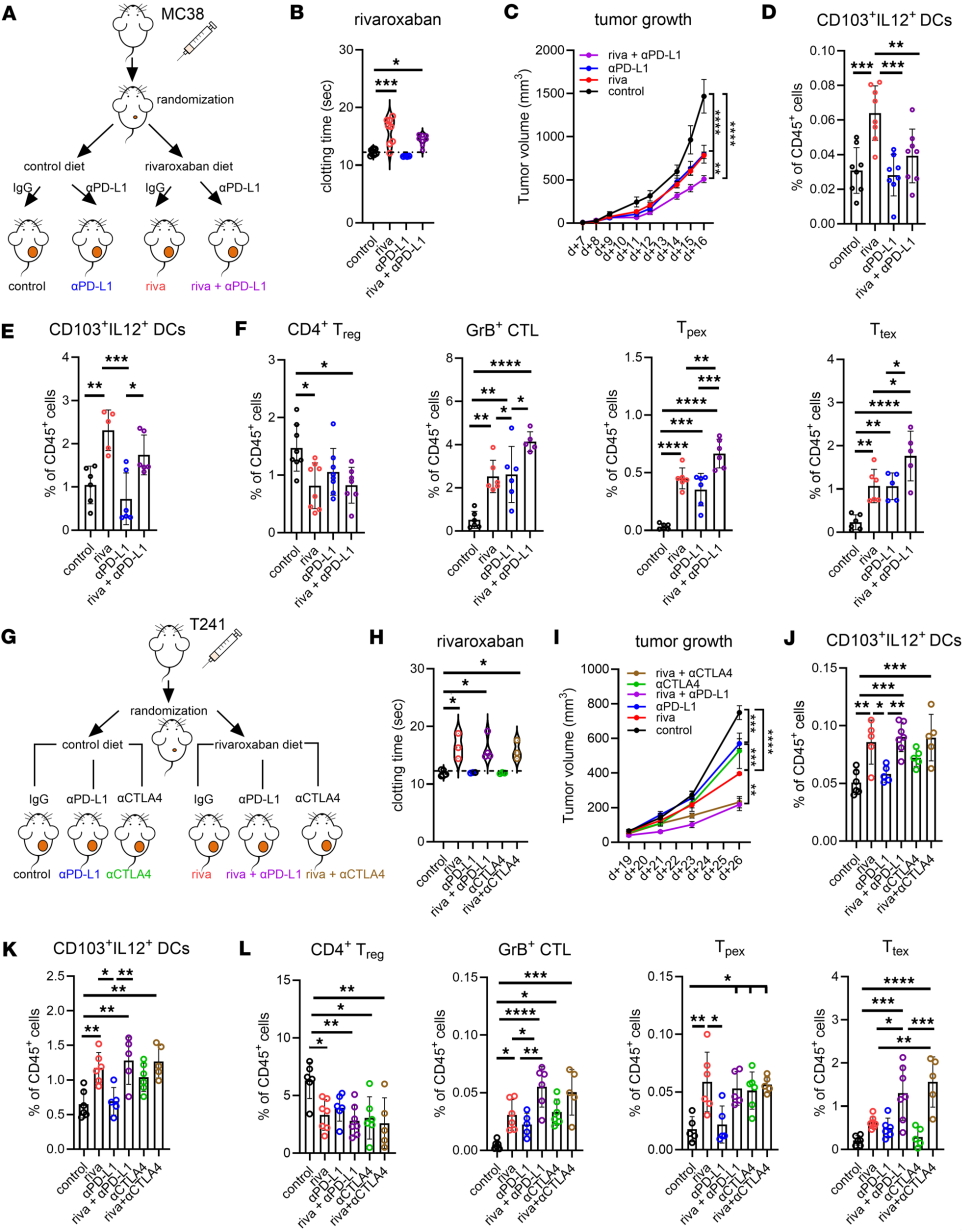
FXa inhibition improves priming function of APCs and synergizes with immune checkpoint blockade to expand progenitor and terminally exhausted CD8^+^ T cells. (**A**) Mice were injected with MC38 tumor cells s.c. in the right flank. Mice were randomized at day 7 into the 4 indicated treatment cohorts. Rivaroxaban was given as a daily diet, αPD-L1 or IgG control was given i.p. every 3 days until sacrifice. (**B**) Rivaroxaban induced prolongation of the prothrombin time at sacrifice; Violin plot, *n =* 4–8, 1-way ANOVA with Sidak’s multiple comparison test. (**C**) Tumor growth curves (*n =* 10–11/cohort); mean SEM, 2-way ANOVA with Tukey’s multiple comparison test. (**D** and **E**) Frequencies of CD103^+^IL12^+^ DCs in the draining LN (**D**) and TME (**E**). (**F**) Frequencies of regulatory T cells (CD4^+^ T_reg_), GrB^+^ CTL, progenitor exhausted (CD8^+^ T_pex_) and terminally exhausted (CD8^+^ T_tex_) CD8^+^ T cells in the TME; *n =* 5–8/group, mean ± SD, 1-way ANOVA with Tukey’s multiple comparison test. (**G**) Mice were injected with T241 tumor cells s.c. in the right flank. Mice were randomized at day 19 into the 6 indicated treatment cohorts. Rivaroxaban was given as a daily diet, αPD-L1, αCTLA4 or IgG control was given i.p. every 3 days until sacrifice. (**H**) Rivaroxaban-induced prolongation of the prothrombin time at sacrifice; Violin plot, *n =* 3–4, 1-way ANOVA with Sidak’s multiple comparison test. (**I**) Tumor growth curves (*n =* 4–5/cohort); mean SEM, 2-way ANOVA with Tukey’s multiple comparison test. (**J** and **K**) Frequencies of CD103^+^IL12^+^ DCs in the draining LN (**J**) and TME (**K**). (**L**) Frequencies of regulatory T cells (CD4^+^ T_reg_), GrB^+^ CTL, progenitor exhausted (CD8^+^ T_pex_), and terminally exhausted (CD8^+^ T_tex_) CD8^+^ T cells in the TME; *n =* 5–8/group, mean ± SD, 1-way ANOVA with Tukey’s multiple comparison test. *P < 0.05; **P < 0.01; ***P < 0.001; ****P < 0.0001.

As demonstrated above, loss of myeloid FX expression or FXa-PAR2 signaling improved tumor debris uptake and APC function by induction of the cGAS-STING-IFN-I axis (Figure 1E and Figure 3) and treatment with the direct FXa inhibitor rivaroxaban attenuated the formation of aggregates of immune-suppressive platelets with monocytes in tumor-bearing mice (Figure 5C). We hypothesized that rivaroxaban’s immunostimulatory effects in the context of checkpoint inhibitor therapy (10, 26) were associated with an expansion of IL-12 expressing CD103^+^ DCs, resulting in improved T cell priming. We first injected MC38 tumor cells into WT mice and randomized mice with detectable tumors into 4 treatment groups (Figure 8A). Rivaroxaban treatment prolonged the clotting time of plasma from tumor-bearing mice irrespective of checkpoint inhibitor αPD-L1 therapy (Figure 8B), demonstrating effective anticoagulation. Rivaroxaban or αPD-L1 both significantly reduced tumor growth and demonstrated synergistic effects when combined (Figure 8C).

To elucidate the synergistic mechanism, we analyzed tumor draining lymph nodes and the TME for the abundance of CD103^+^ IL12^+^ DCs. The frequencies of CD103^+^IL12^+^ DCs in the draining lymph nodes (Figure 8D) and the TME (Figure 8E) were only increased in mice receiving rivaroxaban treatment, whereas αPD-L1 alone showed no effect. As a surrogate marker for improved T cell priming and reactivation, we measured the frequencies of progenitor (T_pex_) and terminally (T_tex_) exhausted CD8^+^ T cells as well as of granzyme B–expressing cytotoxic T cells (GrB^+^ CTL). The abundance of regulatory CD4^+^FoxP3^+^ T cells was reduced specifically in the rivaroxaban treatment groups (Figure 8F). In contrast, an increase in GrB^+^ CTL was observed upon single rivaroxaban or αPD-L1 treatment, but markedly enhanced by the combination therapy. In accordance, progenitor exhausted T_pex_ and terminally exhausted T_tex_ CD8^+^ T cells were also most enriched upon rivaroxaban and αPD-L1 combination therapy (Figure 8F).

We next addressed the question of whether rivaroxaban treatment also synergized with the checkpoint inhibitor αCTLA. In contrast to αPD-L1, which predominantly reverses the suppression of effector T cells, αCTLA mainly acts on the priming phase of T cells in the lymph nodes. We injected WT mice with T241 tumor cells and randomized the mice to mono or combination therapy of αPD-L1 or αCTLA with rivaroxaban (Figure 8G). All mice receiving rivaroxaban treatment had similarly prolonged plasma clotting times (Figure 8H). All drugs significantly reduced tumor growth when used as monotherapy, while the combination of rivaroxaban with both checkpoint inhibitors demonstrated a marked and synergistic reduction of tumor growth in this model (Figure 8I). In contrast to αPD-L1, αCTLA4 treatment alone was already associated with an increased frequency of CD103^+^IL12^+^ DCs in the draining lymph nodes (Figure 8J) and the TME (Figure 8K) compared with the control group. Rivaroxaban and αCTLA4 treatment were also similar in reducing CD4^+^FoxP3^+^ T cell abundance and increasing T_pex_ cells in the TME (Figure 7L). Remarkably, antigen presentation–competent DCs as well as GrB^+^ CTL and the terminally exhausted CD8^+^ T cells (T_tex_) were synergistically expanded by the combination therapy of rivaroxaban with αCTLA4 (Figure 8, J–L). These data demonstrated for different tumor entities that pharmacological intervention with rivaroxaban recapitulated the improved antigen presentation and T cell priming capacity of APCs delineated with myeloid FXa-PAR2 signaling–deficient mice and enhanced the main principles of clinically approved check point inhibitor therapy.

## Discussion

Cancer is a systemic disease with a complex interplay between a diversity of cell types and imposes inflammatory conditions associated with the activation of the coagulation system. At the cellular level, the interactions of platelets with immune and tumor cells are key players, and activation of platelets in malignancy occurs by various stimuli, including podoplanin and thrombin, which facilitate tumor cell metastasis as consequence of tumor cell–platelet aggregate formation (66, 67). This partially relies on thrombin-driven integrin GPIIb-IIIa (CD41/CD61) activation (66), and this integrin also binds prothrombin and contributes to the prothrombinase activity amplifying thrombin generation by FVa/FXa on the platelet surface (68).

Here, we show that the formation of monocyte-platelet aggregates in the TME as well as in the circulating blood is remarkably dependent on monocyte FX expression as well as cell autonomous FXa-PAR2 signaling. In addition, circulating tumor educated platelets show higher expression of GARP and TGFβ/LAP in mice and humans, as demonstrated here and elsewhere (69), and are prone to form monocyte-platelet aggregates that sustain increased local thrombin generation and thrombin-mediated immunosuppressive TGFβ release. This aggregate formation is prevented by clinically used oral anticoagulants, providing a therapeutic strategy to reverse platelet-mediated immunosuppression in cancer progression.

Myeloid cell–autonomous FXa-PAR2 signaling also promotes platelet recruitment and mediates immunosuppressive alterations in the TME by influencing the phenotype of monocyte-derived DCs and macrophages. For effective antitumor immune responses, the induction of the cGAS-STING-IFN-I pathway that is driven by the uptake of tumor-derived nucleic acids plays a major role (46, 47). IFN-I regulates the abundance of TCF1^+^ stem-like CD8^+^ T cells as well as the production of IFN-induced genes, including CXCL10 and Midline 1, which are relevant for the migration and recruitment of CTL into the TME and for the exocytosis of lytic granules, as described in our study and elsewhere (60, 64, 65, 70). Furthermore, cGAS-STING signaling is also crucial for the efficacy of ICB treatment (71) and combination therapies of STING agonists and ICB have been and are currently investigated in clinical trials (NCT03172936; NCT05846659; NCT05846646; NCT05070247).

We show that the presence of platelets, myeloid cell–derived FXa, and thrombin reduces the uptake of tumor cell–derived nucleic acids, while lack of myeloid cell FX expression or FXa-PAR2 signaling conversely increases cGAS-STING-IFN-I induction in vitro and in vivo. These coagulation pathways thus interfere with the priming capacities of DCs and reduce the TME abundance of progenitor-exhausted CD8^+^ T cells required for effective checkpoint inhibitor therapy. In addition to lowering tumor cell–derived nucleic acid uptake, platelets and thrombin also reduce CD103 expression during DC differentiation. This provides a rational explanation why the absence of myeloid cell FX expression or treatment with the FXa inhibitor rivaroxaban, which also limits thrombin generation, increases the abundance of CD103^+^ DCs and shows synergistic effects with ICB in vivo.

The presented data define mechanistic details of coagulation signaling–mediated immunosuppression and identify a convergent function of FXa and thrombin as crucial modulators of APC function in the TME. In consequence, the pharmacological suppression of these immune evasive mechanisms by tissue penetrant oral anticoagulants increases the T cell priming capacity of APCs and the accumulation of progenitor exhausted CD8^+^ T cells that are activated as an essential part of efficient ICB (61). We show that FXa inhibitor treatment synergizes with therapy of both αPDL1 and αCTLA4, which interfere with distinct immune checkpoints, promoting tumor immune evasion. While prospective clinical trials are required, combined treatment with oral anticoagulants may also beneficially influence the potentially increased risk of CAT observed under ICB treatment (8, 72).

Although we demonstrate that anticoagulants such as the direct FXa inhibitor rivaroxaban improves APC function and the priming of T cell responses leading to attenuated tumor growth, we do not know whether these beneficial effects are generally applicable to human cancers with variable immunogenic profiles and whether synergistic effects will be seen also with other treatment combinations, including chemotherapy or radiation, which cause cell death and release of nucleic acids as part of their immunostimulatory activity. Prospective clinical trials with oral anticoagulants, as (neo)adjuvant therapies will need to control such pretreatments and drug interactions, comorbidities, and anatomical location of tumors that may predispose to unwanted bleeding. In addition, biomarker profiles such as FX^+^ monocytes and their aggregate formation with platelets may be required to identify patients who benefit the most from this kind of intervention. This approach will ultimately help to develop strategies for tailored therapy and simultaneous prevention of cancer-associated thrombosis and immunosuppression in specific tumor types.

## Methods

### Sex as a biological variable.

As only female mice can be used in the spontaneous breast cancer model PyMT, we used only female mice for the transplantable tumor models MC38, T241, and PyMT, to make data comparable. For in vitro differentiation assays, male as well as female mice were used as bone marrow donors. Experiments were sex and age matched.

### Study design.

Adult mice of similar age (10 to 12 weeks) and same sex from the same breeding colony were used. Comparisons of PyMT tumor development involved cohorts in the same animal facility to avoid environmental variability. Tumor cell suspensions of transplantable tumors were randomly injected into different lines that originated from littermate founders or were cre-deleter strains of floxed alleles and littermate controls without cre-recombinase. Effects of host genetic mutations were independently confirmed in repeat experiments or different tumor models, and pooled data were analyzed. For treatment experiments, groups were randomized by cage, assuring equal tumor volume at the beginning of treatment. Treatment and tumor monitoring were performed by the same investigators, but analysis of flow cytometry data was performed without knowledge of the treatment groups. Flow cytometry comparisons were based on biological replicates stained with the same fluorophore combinations and compensations analyzed on the same flow cytometer. Expression profiles were obtained from biological replicates randomly analyzed by quantitative polymerase chain reaction (RT-PCR) in parallel; technical failures were eliminated from the analysis.

### Animal models and tumor models.

We used C57BL/6N (*C57BL6/NCrl*; Charles River), F10^fl/fl^ (*B6(Cg)-F10^tm1c(EUCOMM)/Hmgu^/Tarc*), F10^fl/fl^LysMcre (*B6(Cg)-F10^tm1c(EUCOMM)/Hmgu^ Lyz2^tm1(cre)Ifo^/Tarc*), PAR2^WT^ (*C57BL/6(Cg)*), PAR2^G37I^ (*B6(Cg)-F2rl1^tm2.1Wmrf^/Tarc*), PyMT-PAR2^G37I^ (*B6(Cg)-Tg^(MMTV–PyVT)634Mul/LeIIJ^ F2rl1^tm2.1Wmrf^/Tarc*), PyMT-PAR2^WT^ (*B6(Cg)-Tg^(MMTV–PyVT)634Mul/LeIIJ^/Tarc*), PyMT-F10^fl/fl^LysMcre (*B6(Cg)-Tg^(MMTV–PyVT)634Mul/LeIIJ^ F10^tm1c^ ^(EUCOMM)/Hmgu^ Lyz2^tm1(cre)Ifo^/Tarc*), and PyMT-F10^flfl^ (*B6(Cg)-Tg^(MMTV–PyVT)634Mul/LeIIJ^ F10^tm1c^ ^(EUCOMM)/Hmgu^/Tarc*) mice (in-house breeding). In the spontaneous breast cancer model, PyMT, first, palpable tumors usually develop at an age of 9–10 weeks in female mice. Mice were usually sacrificed at an age of 19–20 weeks, when tumors reached maximum approved size. Transplantable tumor models,MC38 (kind gift from Bohn T, FZI, University Medical Center, Mainz, Germany), T241 (Ruf laboratory, La Jolla, California, USA; (73)) or PyMT (own established cell line) were s.c. injected into the right flank (5 x 10^4^ cells in 100 μL PBS). Rivaroxaban, provided by S. Heitmeier (Bayer AG, Leverkusen, Germany), was formulated at a concentration of 0.4 mg/g of chow from SNIFF (Soest, Germany), and cohorts were fed the drug formulation or a control diet from the same manufacturer. The checkpoint inhibitors αPD-L1 (10F.9G2) or αCTLA4 (9D9) were given 3 times intraperitoneally at a dose of 100 μg/mouse with 3 days intervals.

Tumor scoring was performed as previously published (26). PBMCs were separated by FICOLL from peripheral blood. Single cell suspensions of minced and digested tumors (2 mg/mL collagenase A, 5 U/mL DNaseI in DMEM; see Supplemental Table 1) or of draining lymph nodes (1 mg/mL collagenase D in DMEM) were passed through 40 μm cell strainers, resuspended in PBS, stained with the respective antibodies, and analyzed by flow cytometry.

### Flow cytometry.

Cells directly isolated from mouse tissues or from culture plates were incubated with Fc-block (αCD16/32) in PBS/0.5% BSA for 15 min at 4°C before staining. Cells were washed once. For surface epitopes, cells were stained for 20 min at 4°C with the antibody cocktail, as indicated in the respective experiment. For intracellular epitopes, cells were fixed and permeabilized using the Foxp3 / Transcription Factor Staining Buffer Set (eBioscience). The intracellular antibodies were diluted in the permeabilization buffer. Cells were analyzed on an Attune NxT (Thermo Fisher) and data were analyzed with FlowJo10.8.1. Antibodies were purchased from eBioscience, BD or biolegend (Supplemental Table 1).

### scRNA-seq of TAMs and tumor infiltrating lymphocytes.

CD11c bead selected cells from late-stage tumors or CD4/CD8 bead selected cells from mid-size tumors were frozen in 90% FCS/10% dimethyl sulfoxide and thawed cell suspensions were processed for single cell sequencing on the 10x Genomics platform as described (26). Three independent biological replicates per genotype and cell type were sequenced and analyzed as described (26, 74).

### Immunohistochemistry.

PFA fixed human pancreatic cancer samples were stained with αCD45, αCD68, and αFX (Supplemental Table 1). FX^+^CD68^+^ cells within total CD45^+^ cells were determined.

### Western blotting.

Cells were lysed with RIPA lysis buffer and sample concentration adjusted to the desired value. After addition of Laemmli buffer with β-mercaptoethanol, samples were run on a 4%–20% Mini Protean TGX gel (Cat# 45611094). The proteins were transferred to a 0.2 μm PVDF membrane (Cat#1704156) with the Trans Blot Turbo System from BioRad. Membranes were blocked with milk powder TBST and incubated over night with anti-mouse prothrombin (abcam AB208590; 1:1000), anti-mouse FX (Ruf lab; 1:500), anti-mouse FV(a) (GMA-753 Green Mountain; 1:1000), anti-mouse IRF3 (D83B9, #4302, Cell Signaling; 1:1000), anti-mouse phospho-IRF3 (Ser396) (4D4G, #4947, Cell Signaling; 1:1000), and anti-mouse β actin (8H10D10, #3700, Cell Signaling; 1:1000) in 5% dry milk-TBST, washed and incubated with the secondary antibody anti-rabbit HRP conjugated (# 7074, Cell Signaling, 1:5000) or anti-rat HRP (# 7077 Cell signaling, 1:5000), and finally developed with ECL reagent (SIRIUS).

### In vitro differentiation of moDCs and macrophages.

Monocytes were isolated from bone marrow using the EasySep monocyte isolation kit (Stem cell) according to manufacturer instructions. Monocytes were plated (2x10^5^/well) in 12 well plates and cultured in serum free X-Vivo 15 (Lonza) supplemented with vitamin K (Konakion, 80 ng/mL). M-CSF (20 ng/mL) was added for macrophage differentiation and GM-CSF (20 ng/mL) + IL4 (20 ng/mL) for immature DC differentiation. For maturation of DCs, a maturation cocktail (TNF 10 ng/mL, IL1β 10 ng/mL, PG_E2_ 1 μg/mL, IL6 10 ng/mL) was added for another 2 days.

### Real-time PCR.

Total RNA was extracted with Trizol, and cDNA was synthesized from 100 ng of total RNA with LunaScript RT SuperMix (New England Biolabs). Relative expression levels were determined by RT-PCR on a BioRad Real-Time System (CFX Connect Real-Time System; CFX96 Real-Time System) using Luna Universal qPCR Master Mix (New England Biolabs). Primer sequences are listed in Supplemental Table 2. r18s was used for normalization, and standard curves for each target gene were generated by pooled cDNA.

### Isolation of platelets.

Citrated blood was taken from healthy or tumor bearing mice. Platelets were separated as published (75) and resuspended in X-Vivo 15 for further in vitro use.

### Active TGFβ levels in cell culture supernatant.

Cell culture supernatant from in vitro generated macrophages was collected at day 5 after macrophages were either or not exposed to platelets in a ratio of 1:10 in prothrombin supplemented medium. TGFβ levels were determined with the TGF beta1 mouse ELISA (enzyme linked immunosorbent assay) Kit (Abcam, cat#ab119557).

### Cytokine release by platelets.

Platelets (9x10^6^ in 100 μL X-Vivo 15) were either used untreated or activated with thrombin (4 U/1000 μL) for 15 min. Platelet free supernatant was collected after centrifugation and measured for active TGFβ content using a Legendplex kit (Biolegend). Data was analyzed with the software provided by the manufacturer.

### Thrombin generation.

In vitro generated macrophages or DCs (2x10^5^/well) were stimulated over night with LPS (1 μg/mL) and IFNγ (100 ng/mL) at day 5 of culture to stimulate FX expression and activation. Freshly isolated platelets (2x10^6^/well) were added as indicated in the respective experiment in serum free X-Vivo 15 supplemented with prothrombin (200 nM). Cells were incubated for 15 min to allow prothrombin to thrombin conversion. Supernatants were quenched in 50 mM TRIS, 150 mM NaCl, pH 8.3 containing 400 nM Rivaroxaban. Subsequently, thrombin activity was measured with pNAPEP-0238 (Cryopep) in a Spectramax kinetic plate reader.

### Uptake of EdU labeled tumor debris.

MC38 was cultured in RPMI/10%FCS until 50% confluency. EdU labeling was performed using the Click-iT Plus EdU Alexa Fluor 594 Flow Cytometry Kit as published (Invitrogen) (56). EdU containing reagent (final: 10 μM) was added to the cells. Cells were allowed to incorporate EdU for up to 24 hours in their DNA. Cells were washed with PBS, harvested, resuspended in serum free RPMI (2x10^6^/mL), and heat shocked at 55°C for 60 min with agitation every 15 min to break them up. Tumor debris was centrifuged at 300*g* for 10 min to remove cell fragments. Cell free supernatant was stored in aliquots at -20°C until usage.

### cGAMP ELISA.

For quantification of intracellular cGAMP in macrophages, BM monocytes were seeded at 3.5 x 10^5^ cells/well in a 12-well plate and differentiated into macrophages as described above. At day 5, cells were loaded for 6 or 16 hours with tumor debris of 2.5 x 10^6^ PyMT tumor cells. Cells were washed three times with PBS and lysed using 200 μL M-PER buffer (Thermo Fisher Scientific) including phosphatase inhibitor and protease inhibitor (Roche). The protein content of lysates was quantified using BCA. The concentration of cGAMP was measured using a cGAMP ELISA (Cayman Chemical) according to the manufacturer’s instructions using 5 μg protein per sample.

### Statistics.

Statistical analyses were performed with GraphPad Prism 10 or R statistical software (74). *P* values of less than 0.05 were considered significant. Statistical comparisons were performed using 2 tailed, unpaired Student’s *t* test for comparing 2 different groups. For more than 2 groups, 1-way ANOVA with Dunnett’s multiple comparison, or 2-way ANOVA with Sidak’s or Tukey’s multiple comparison was used as indicated.

### Study approval.

All animal experiments had approved protocols at Scripps Research (IACUC 09-0111) or University Medical Center Mainz (Landesuntersuchungsamt Koblenz, AZ 23 177-07/G14-1-041; AZ 23 177-07G20-1-081). PBMC samples from healthy donors and lung cancer patients were collected from citrated blood by FICOLL after informed consent (Landesärztekammer Rheinland-Pfalz, ethical approval 2020-15203).

### Data availability.

Single cell sequencing data are available through the NCBI Gene Expressing Omnibus (accession number, GSE214518). All mouse strains are available through a simple academic material transfer agreement, where necessary for institutional transfer.

Processed scRNA-seq data (in the RDS binary format) are available under https://www.ncbi.nlm.nih.gov/geo/query/acc.cgi?acc=GSE214518 and at [zenodo | figshare], and can readily be explored using iSEE (74), an interactive user interface implemented with RStudio’s Shiny (74) (for TILs: http://shiny.imbei.uni-mainz.de:3838/iSEE_PAR2_TILs/; for TAMs: http://shiny.imbei.uni-mainz.de:3838/iSEE_PAR2_TAMs/, https://github.com/federicomarini/PAR2_TAMs_TILs/blob/main/shinyserver_config/iSEE_PAR2_TAMs/app.R). A detailed description of the bioinformatics analysis steps can be found at https://github.com/federicomarini/PAR2_TAMs_TILs All values underlying the data presented in the graphs and as means are available in the Supporting Data Values file.

## Author contributions

PW design and execution of experiments; JP, SP, JR, CW, AH, and MMG execution of experiments; FM and SP, bioinformatics; SR, generation of mouse strains; HW, single cell transcriptomics; TK, collection of patient samples; TM, reviewed and edited the manuscript; CG, study conception, design and execution of the experiments, data analysis and interpretation, preparation of figures, and writing of the manuscript; WR, study conception, design and supervision, data analysis and interpretation, and writing of the manuscript.

## Supplementary Material

Supplemental data

Unedited blot and gel images

Supporting data values

## Version 1 07/22/2025

In-Press Preview

## Version 2 09/09/2025

Electronic publication
