# Supplementary material for: Coagulation proteases modulate nucleic acid uptake and cGAS-STING-IFN induction in the tumor microenvironment
Source: JCI Insight. 2025 Jul 22;10(17):e190311. doi: 10.1172/jci.insight.190311 (PMC12487691; doi:10.1172/jci.insight.190311)
Supplement: Supplemental data [file jciinsight-10-190311-s225.pdf]

Coagulation proteases modulate nucleic acid uptake and cGAS-STING-IFN induction in the tumor microenvironment

Petra Wilgenbus[1], Jennifer Pott[1], Sven Pagel[1], Claudius Witzler[1], Jennifer Royce[2], Federico Marini[3,4,5], Sabine Reyda[1], Thati Madhusudhan[1], Thomas Kindler[6,7,8], Anne Hausen[9], Matthias M. Gaida[4,8,9], Hartmut Weiler[10], Wolfram Ruf[1,2,4,5*], Claudine Graf[1,4,5*]

[1] Center for Thrombosis and Hemostasis, Johannes Gutenberg University Medical Center, Mainz, Germany

[2] Department of Immunology and Microbiology, Scripps Research, La Jolla, CA, USA

[3] Institute of Medical Biostatistics, Epidemiology and Informatics (IMBEI), Johannes Gutenberg University Medical Center, Mainz, Germany

[4] Research Center for Immunotherapy (FZI), Johannes Gutenberg University Medical Center, Mainz, Germany

[5] Center for Translational Vascular Biology (CTVB), Johannes Gutenberg University Medical Center, Mainz, Germany

[6] University Cancer Center and 3rd Medical Department, Johannes Gutenberg University Medical Center, Mainz, Germany

[7] German Cancer Consortium (DKTK), partner site Frankfurt/Mainz, a partnership between DKFZ and University Medical Center Mainz, Germany

[8] TRON, Translational Oncology at the University Medical Center, Johannes Gutenberg University, Mainz, Germany

[9] Department of Pathology, Johannes Gutenberg University Medical Center, Mainz, Germany

[10] Versiti Blood Research Institute and Medical College of Wisconsin, Department of Physiology, Milwaukee, WI, USA

* Corresponding authors:

Claudine Graf (grafc@uni-mainz.de) and Wolfram Ruf (ruf@uni-mainz.de)

Supplemental Materials

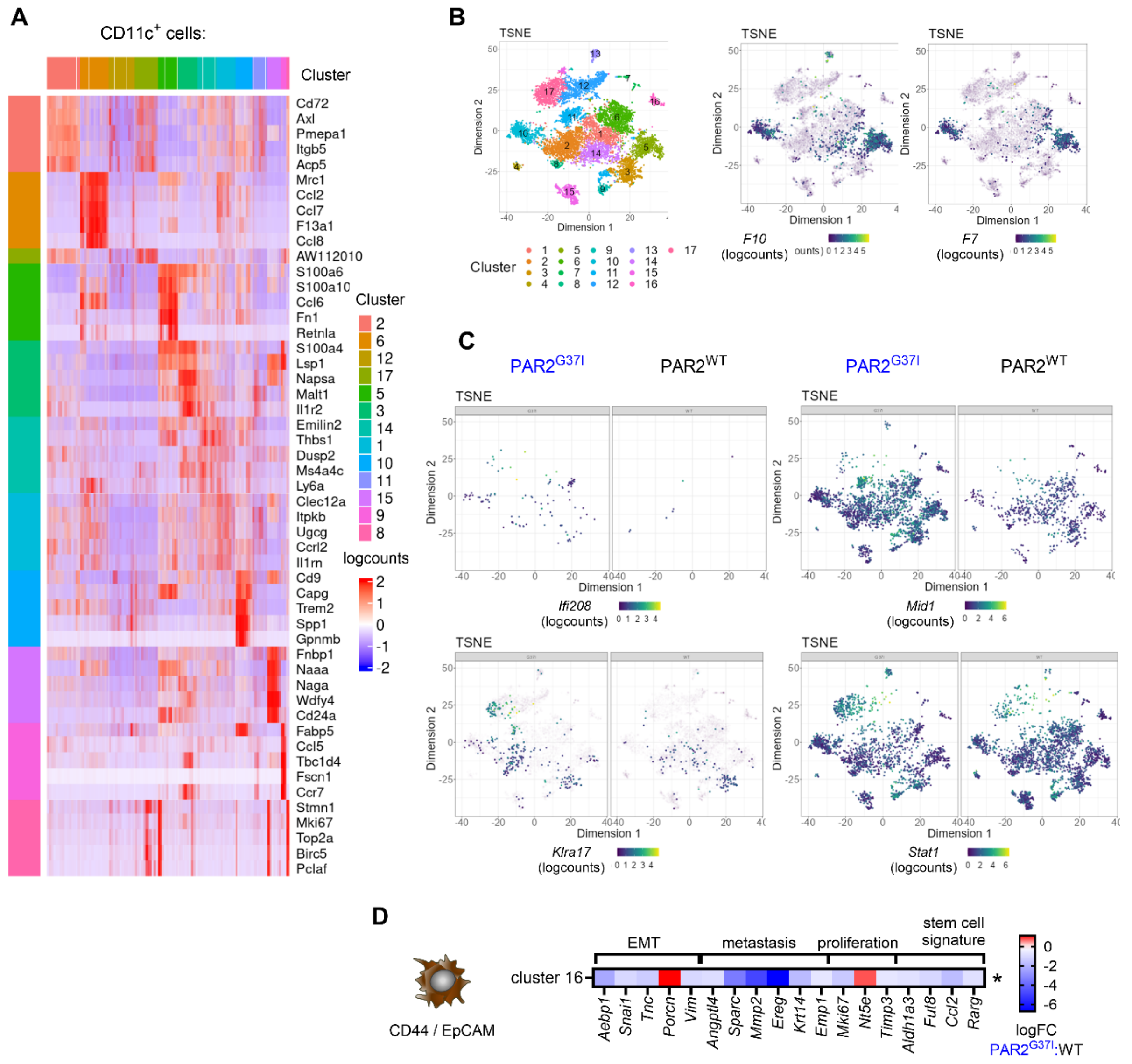

Supplemental Figure 1: Single cell RNA-seq characterization of CD11c-selected cells from the TME of PyMT mice. Shown are merged data sets from CD11c-selected cells from the TME of PyMT-PAR2^{WT} and PyMT-PAR2^{G37I} mice. (**A**) The top five highly expressed cluster discriminating markers in the merged data set determined by comparison of each cluster against the combined data from the other clusters. (**B**) tSNE plots of the cluster assignments and the expression patterns of *F10* and *F7* in the clusters shown in Figure 1A. (**C**) tSNE plots showing the expression patterns of the IFN inducible genes *Ifi208, Mid1, Klra17* and *Stat1* for each genotype. (**D**) Differential transcript abundance between the genotypes of genes relevant for epithelial to mesenchymal transition (EMT), metastasis, proliferation, and stem cell signature in *Cd44/Epcam*$^+$ tumor cells (cluster 16); n = 3; *p_adj.loc<0.05.

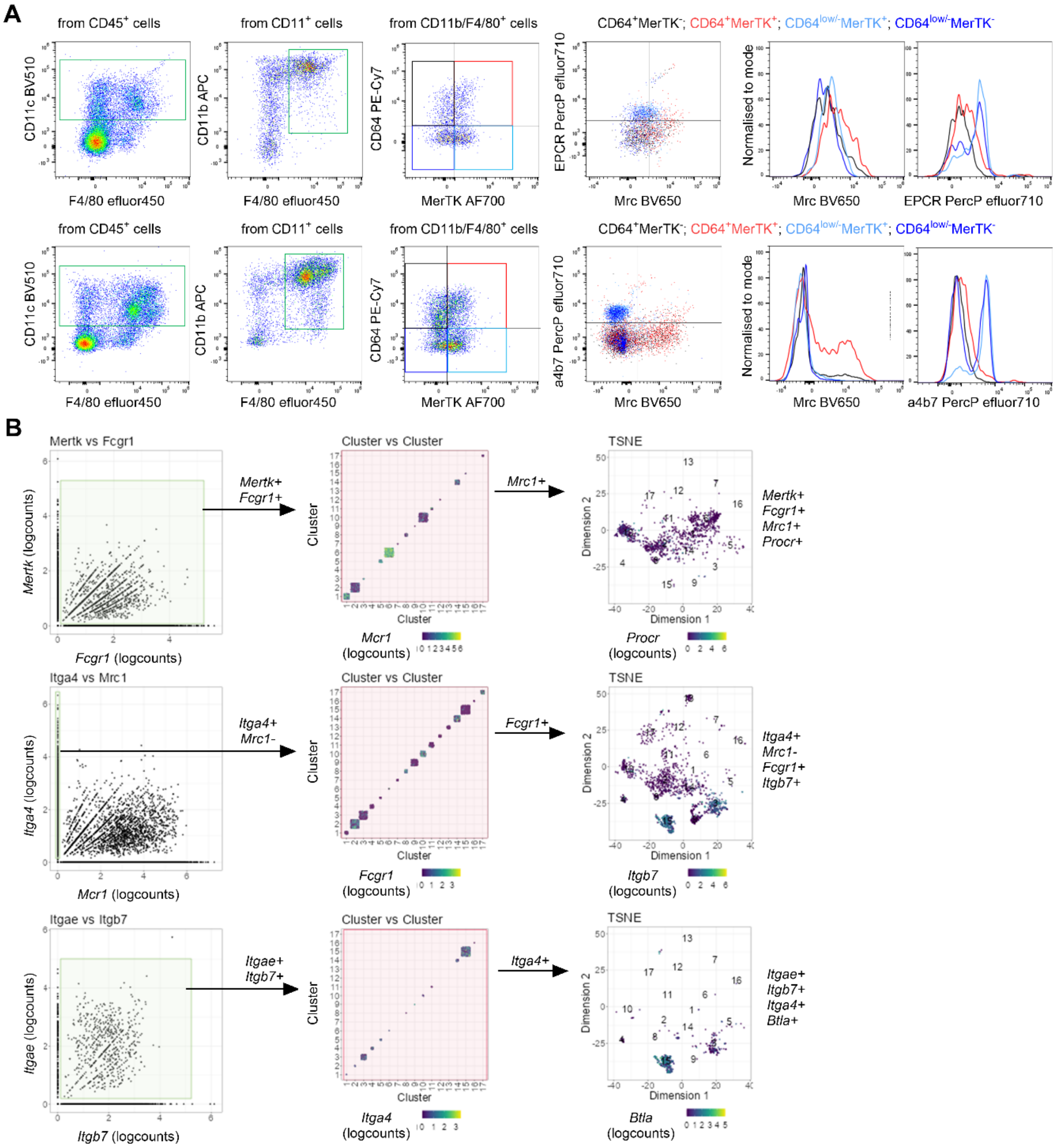

Supplemental Figure 2: Characterization of macrophages and DCs by flow cytometry. (**A**) Gating strategy used for quantification of macrophage subpopulations by flow cytometry in CD45$^+$ cells from the TME of PyMT-F10$^{fl/fl}$ and PyMT-F10$^{fl/fl}$LysMcre mice (see Figure 2A for analysis). (**B**) Transcript levels of markers to characterize macrophage and DC subsets by flow cytometry. Co-expression of macrophage markers in the merged scRNA-seq data set of PyMT-PAR2^{G37I} and PyMT-PAR2^{WT} mice were used to identify surface markers. Cells expressing the transcripts of the marker combinations were first selected, as indicated by the green boxes (left panels), and then analyzed for expression levels of the third maker in each cluster (middle panels). Selected cells were then mapped onto the tSNE plots (third panels) with display of the expression levels of the 4th marker.

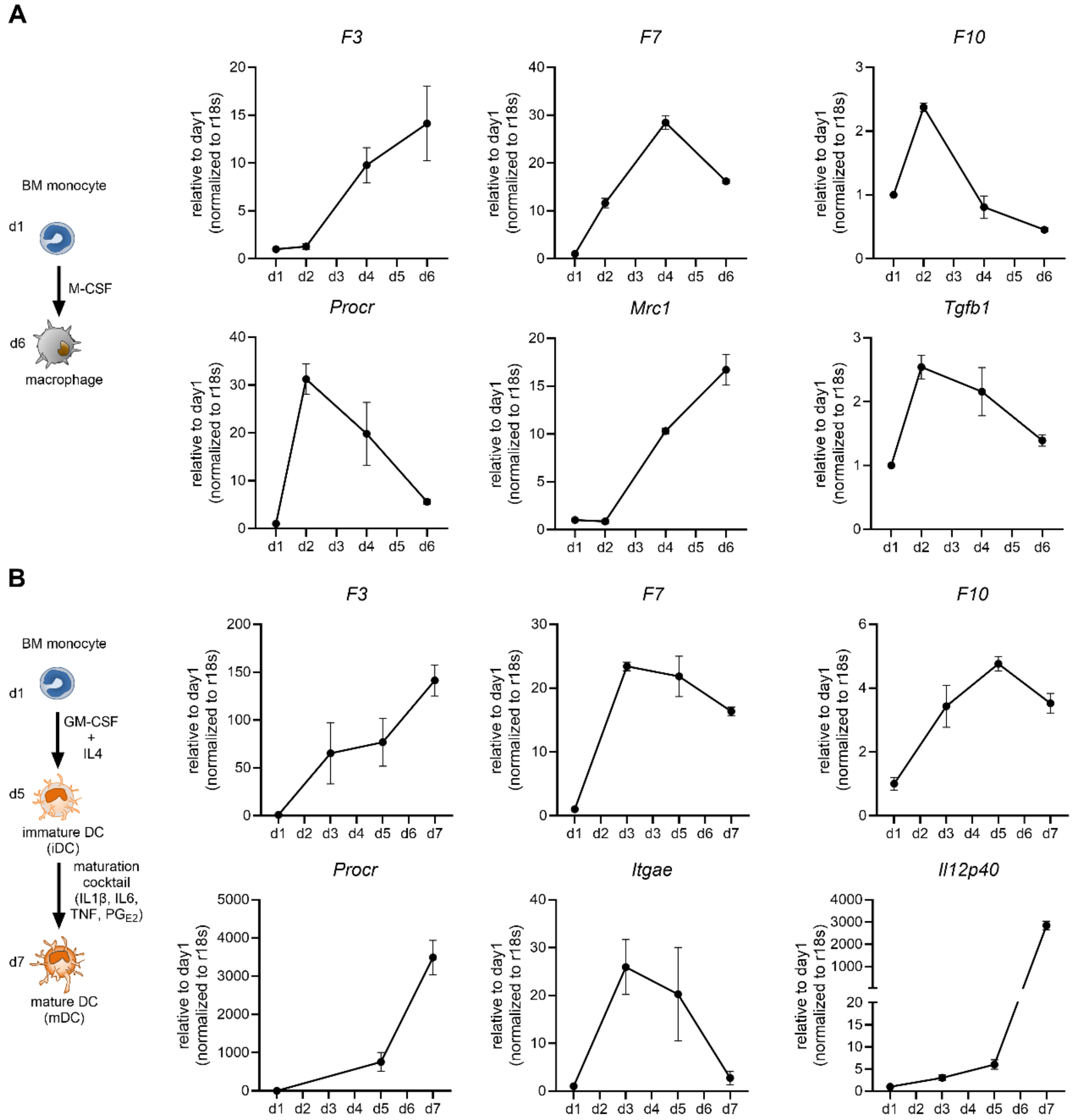

Supplemental Figure 3: Characterization of macrophages and DCs in vitro. (**A**) mRNA expression of coagulation factors and macrophage markers normalized to r18s during differentiation of BM monocytes from C57/BL6 mice with M-CSF into macrophages; mean ± SD, n = 3. (**B**) mRNA expression of coagulation factors and DC markers normalized to r18s during differentiation of BM monocytes from C57/BL6 mice with GM-CSF and IL4 into iDCs. For maturation, iDCs were exposed on day 5 to IL1β/TNF/IL6/PG_{E2} for additional two days; mean ± SD, n = 3.

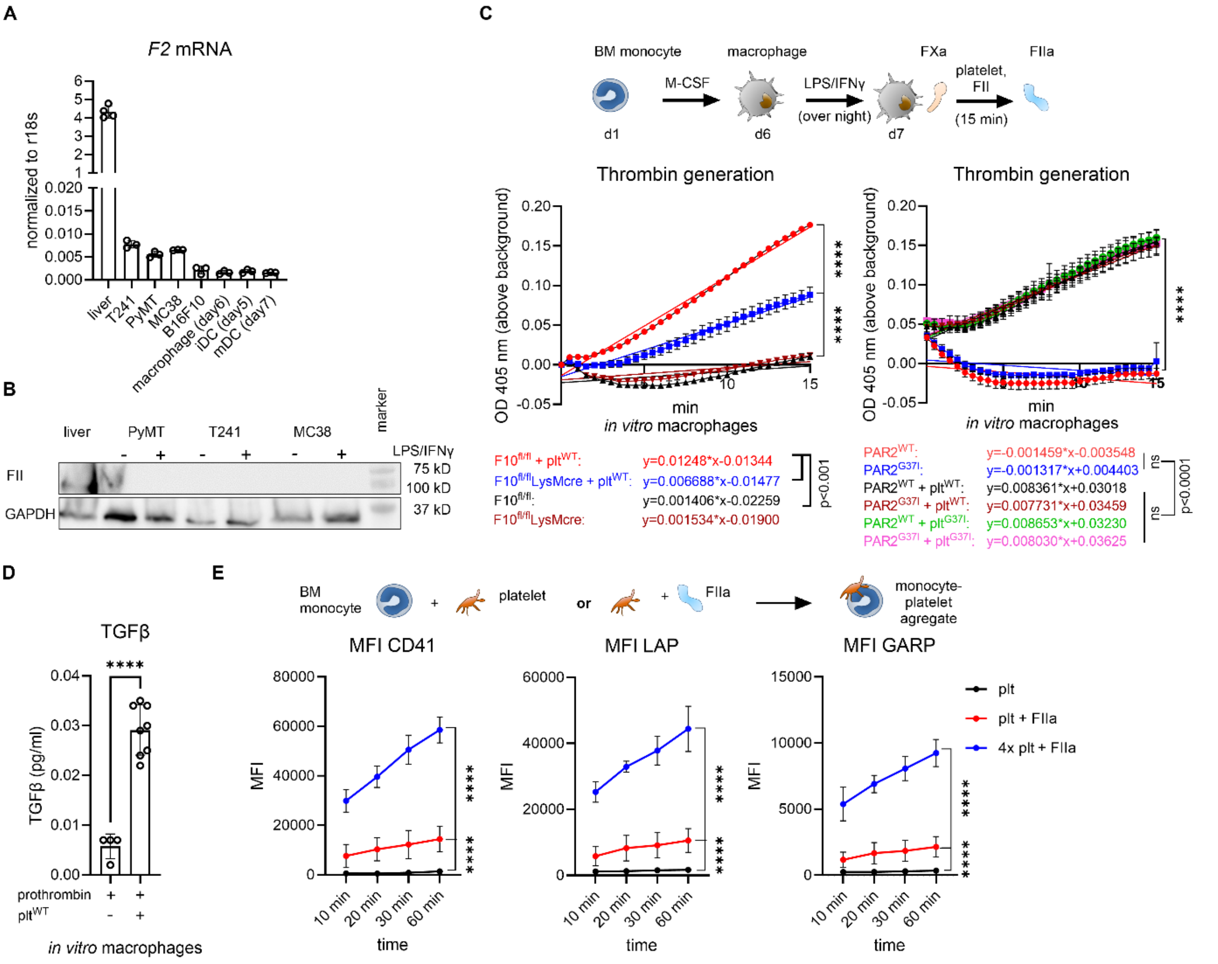

Supplemental Figure 4: Prothrombin expression by tumor cell lines and FXa dependent platelet interactions. (**A**) *Prothrombin* (*F2*) mRNA expression by liver, the tumor cell lines T241, PyMT, MC38, and B16F10 and of in vitro differentiated macrophages, iDCs and mDCs normalized to r18s; n=3. (**B**) Western blot analysis of prothrombin expression by the tumor cell lines PyMT, MC38 and T241 with and without LPS/IFNγ stimulation overnight. Liver was used as a positive control. (**C**) Thrombin generation by in vitro differentiated macrophages from F10^{fl}LysMcre or F10$^{fl/fl}$ mice, stimulated over night with LPS/IFNγ to enhance FX expression and with or without subsequent addition of WT platelets at a macrophage to platelet ratio of 1:10. Thrombin generation of in vitro differentiated macrophages from PAR2^{WT} or PAR2^{G37I} mice was analyzed analogously in the presence or absence of platelets from a PAR2^{WT} (plt^{WT}) or PAR2^{G37I} (plt^{G37I}) mice; n=3, mean ± SD, two-way ANOVA with Tukey's multiple comparison test and simple linear regression analysis. (**D**) Quantification of active TGFβ release into supernatant of in vitro differentiated WT macrophages exposed to prothrombin with or without WT platelets; n=4-8, mean ± SD, two-sided unpaired t-test. (**E**) Accumulations of CD41, LAP and GARP on the surface of WT BM monocytes after incubation with WT platelets in the presence or absence of thrombin for the indicated times slots; n=3, mean ± SD, two-way ANOVA with Tukey's multiple comparison test and simple linear regression analysis.

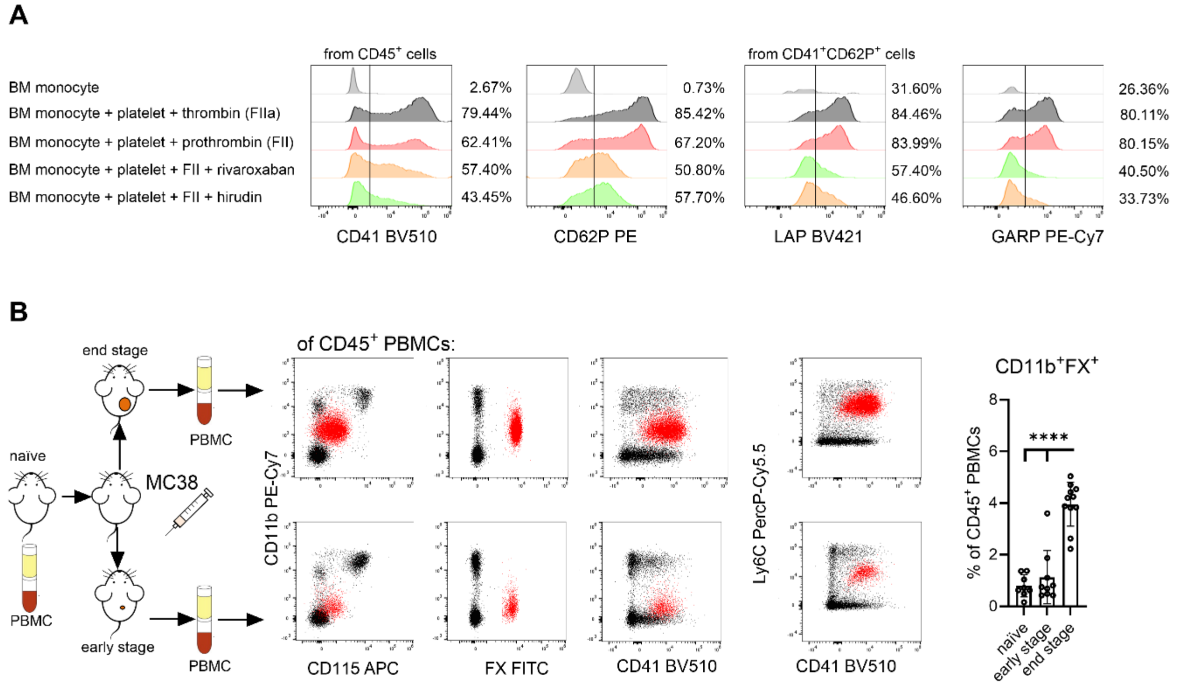

Supplemental Figure 5: Effects of protease inhibitors on monocyte-platelet interactions. (**A**) Histograms and quantification of CD41, LAP and GARP levels on the surface of WT BM monocytes after incubation with WT platelets in the presence of the indicated proteases and the FXa inhibitor rivaroxaban or the thrombin inhibitor hirudin. Histograms show representative examples of n=4. (**B**) Frequency of FX expressing monocytes in the peripheral blood of MC38 tumor bearing WT mice at early or late stages of tumor progression compared to naïve control mice; n=8-11, mean ± SD, one-way ANOVA with Dunnett's multiple comparison test. ****P<0.0001.

Supplemental Table 1: Antibodies and material used

Reagent or Resource	Source	Identifier
Antibodies, anti-mouse		
CD4/CD8(TIL) micro beads	Miltenyi Biotec	Cat# 130-116-480
CD11c Microbeads UltraPure	Miltenyi Biotec	Cat#130-108-338
fixable viability dye, APC efluor780	ebioscience	Cat#65-0865-18, RRID: n.a.
CD3-AF700, 17A2	ebioscience	Cat#56-0032-82; RRID: AB_529507
CD3ε-APC, 145-2C11	ebioscience	Cat#17-0031-82; RRID: AB_469315
CD4-BV650, GK1.5	Biolegend	Cat#100555; RRID: AB_2562529
CD8α-efluor506, 53-6.7	ebioscience	Cat#69-0081-82: RRID:AB_2637161
CD11b-APC, M1/70	ebioscience	Cat# 17-0112-82; RRID:AB_469343
CD11b-BV650, M1/70	BD	Cat#563402; RRID: AB_2738184
CD11b-PE-Cy7, M1/70	ebioscience	Cat# 25-0112-82; RRID:AB_469588
CD11c-BV510, N418	Biolegend	Cat#117353, RRID: AB_2686978
CD11c-BV650, N418	Biolegend	Cat#117339, RRID: AB_2562414
CD19-AF700, eBio1D3	ebioscience	Cat# 56-0193-82; RRID:AB_837083
CD19-APC, eBio1D3	ebioscience	Cat# 17-0193-82; RRID:AB_ 1659676
CD41-BV510, MWReg30	Biolegend	Cat#133923, RRID:AB_2564013
CD44-efluor450, IM7	ebioscience	Cat# 48-0441-82; RRID:AB_1272246
CD45-BV605, 30-F11	ebioscience	Cat# 406-0451-82; RRID:AB_2937166
CD62L-AF700, MEL-14	ebioscience	Cat# 56-0621-82; RRID:AB_494003
CD64-PE-Cy7, X54-5/7.1.1	Biolegend	Cat# 139313; RRID:AB_2563903
CD69-efluor450, H1.2F3	ebioscience	Cat# 48-0691-82; RRID:AB_10719430
CD103-efluor450, 2E7	ebioscience	Cat# 48-1031-82; RRID:AB_2574033
CD115-APC, AFS98	ebioscience	Cat# 17-1152-82; RRID:AB_1210789
CD137(4-IBB)-APC, 17B5	Biolegend	Cat# 17-1371-82; RRID:AB_2573162
CD206 (Mrc1), BV650, C068C2	Biolegend	Cat#141723, RRID: AB_2562445
α4β7-PercP-efluor710, DATK32	ebioscience	Cat# 46-5887-82; RRID:AB_2573793
BTLA(CD272)-PercP-efluor710, 6F7	ebioscience	Cat# 46-5950-82; RRID:AB_2573813
CD201 (EPCR)-PercP-efluor710, eBio1560	ebioscience	Cat#46-2012-80; RRID:AB_10718383
CD201 (EPCR)-PE, eBio1560	ebioscience	Cat#12-2012-82, RRID: AB_914317
CXCR5-SB645, MU5UBEE	ebioscience	Cat#64-9185-42, RRID:AB_2724067
F4/80-PE, BM8	ebioscience	Cat#12-4801-82; RRID:AB_465923
F4/80-efluor 450, BM8	ebioscience	Cat#48-4801-82, RRID: AB_1548747
FoxP3-PE, FJK-16s	ebioscience	Cat#12-5773-82, RRID: AB_465936
FX-FITC	Innovative Research	Cat#ISHAMSFXGFFITC100UG
GARP-PE, YGIC86	ebioscience	Cat# 12-9891-82; RRID:AB_1963598
GARP-PE-Cy7, GIC86	ebioscience	Cat# 25-9891-82; RRID:AB_2573562
GrB-PE, NGZB	ebioscience	Cat#12-8898-82, RRID: AB_10870787
IL12-FITC, C15.6	BD	Cat# 560564, RRID: AB_1645243
LAP-efluor450, FNLAP	ebioscience	Cat# 48-9829-42, RRID:AB_2574133
LAP-PE-Cy7, FNLAP	ebioscience	Cat# 25-9829-42, RRID:AB_11043600

Ly108(slamf6)-APC, eBio13G3-19D	ebioscience	Cat# 17-1508-82, RRID:AB_10717668
Ly6C, PercP-Cy5.5, HK1.4	ebioscience	Cat#45-5932-82, RRID: AB_2723343
Ly6G(IA8)-AF700	ebioscience	Cat# 56-9668-82, RRID:AB_2802355
Ly6G, APC, 1A8	ebioscience	Cat#17-9668-82, RRID: AB_2573307
MerTK-AF700, DS5MMER	ebioscience	Cat# 56-5751-82, RRID:AB_2784771
MHC II (I-A/I-E)-AF700, M5/114.15.2	ebioscience	Cat#56-5321-82; RRID:AB_494009
MHCII-PE, M5/114.15.2	ebioscience	Cat# 12-5321-82, RRID:AB_465928
NK1.1-AF700, PK136	ebioscience	Cat# 56-5941-82, RRID:AB_2574505
NK1.1-APC, PK136	ebioscience	Cat# 17-5941-82, RRID:AB_469479
PD1-PercP-efluor710, J43	ebioscience	Cat# 46-9985-82, RRID:AB_11150055
Tcf7/Tcf1-AF488, S33-966	BD	Cat# 567018, RRID:AB_2916388
Tim3-PE-Cy7, RMT3-23	ebioscience	Cat# 25-5870-82, RRID:AB_2573483
Antibodies, anti-human		
CD11b-BV510, ICRF44	BD	Cat#563088, RRID:AB_2737996
CD14-efluor450, 61D3	ebioscience	Cat#48-0149-42, RRID:AB_1272050
CD16-SB645, eBioCB16	ebioscience	Cat#64-0168-42, RRID:AB_2688210
CD41-PercP-efluor710, HIP8	ebioscience	Cat#46-0419-42, RRID:AB_11219870
CD45-AF700, 2D1	ebioscience	Cat#56-9459-42, RRID:AB_2574511
LAP-APC, FNLAP	ebioscience	Cat#17-9829-42, RRID:AB_2573316
FX(f21-4.2)-AF488	in house	https://doi.org/10.1182/blood.2019001530
Histology, anti-human		
CD68/SR-D1 (KP1)	Novusbio	Cat#NB100-683, RRID:AB_2074852
CD45, 2B11	Agilent Dako	Cat#GA751
FX	Novusbio	Cat#NBP1-33320, RRID:AB_2246637
Anti-mouse antibodies for western blotting		
prothrombin	abcam	Cat# AB208590
FX	in house	https://doi: 10.1126/sciimmunol.aaw8405
FV(a)	Green Mountain	Cat#GMA-753
IRF3	Cell Signaling	Cat#4302
Phosphor-IRF3 (Ser396)	Cell Signaling	Cat#4947
β actin	Cell Signaling	Cat#3700
secondary antibodies for western blot		
anti-rabbit HRP	Invitrogen	Cat#31460
anti-rat HRP	Invitrogen	Cat#31470
Chemicals, Peptides, and Recombinant Proteins		
anti-mouse PD-L1 (10F.9G2)	Bio X cell	Cat#BE0101, RRID: AB_10949073
anti-mouse CTLA4 (CD152) (9D9)	Bio X cell	Cat#BE0164, RRID: AB_10949609
rat anti-mouse IgG2b (LTF-2)	Bio X cell	Cat#BE0090, RRID: AB_1107780
citrate concentrated solution (4% (w/v))	Sigma-Aldrich	Cat#S5770-50ml
collagenase A	Roche	Cat#33278523
hirudin recombinant from yeast	Sigma Aldrich	Cat#94581-1EA
Konakion MM 10 mg	CHEPLAPHARM	PZN -04273031
prothrombin, human	Immbiomed	Cat# ADG417
rivaroxaban	Selleckchem	Cat# S3002
serum-albumin, bovine	VWR, Sigma	Cat#A7030-100G

pNAPEP-0238	Cryopep	Cat# 61010238
IL4	PeproTech	Cat#AF-214-14
GM-CSF	PeproTech	Cat#315-03
M-CSF	PeproTech	Cat#315-02
TNF	PeproTech	Cat#315-01A
PG_{E2}	Biogems	Cat#3632464
IL1β	PeproTech	Cat#211-11B
PG_{E1}	Cayman	Cat#13010
IFNγ	PeproTech	Cat#315-05
LPS from salmonella abortus equi	Enzo	Cat#ALX-581-009-L002
poly I:C	Sigma Aldrich	Cat#P0913-50mg

Supplemental Table 2: RT-PCR primer

	forward	reverse
F2	CTGGTTATAAAGGGCGGGTGAC	GCCAGCACAGAACATGTTGTCAG
F3(TF)	CTTATCGGAAAGGCTCAAGCAC	CCAGGAAACTCTTCCATTGCTCG
F7	CCGTCTCCCCGTAGCTGCCT	TGCGGCACAATTCACGTGTCCT
F10	TTCCGGATGAACGTGGCCCCT	ATGCGTGCGTCCAAAACCGCT
Procr	CTGGTGTGGCCGTGGGCATC	TGGGGGAGTCTGTTTGGCGTCA
Tgfb1	GACTTTTCCGCTGCTACTGC	AATAGGGGCGTCTGAGGAAC
Itgae	CCTCCTGGTCTTGGTTGTGATTATAGC	CTAATCTTGGAGCAGACTGTCAGCCTTC
Mb21d1	CTTTTGGAACAGTTGAAAAAAGAGTTTCAAG	GGCACTCAAGAAAGAATGCTAACAAC
Sting1	CTGTTTGCCATGTCACAGGATG	CCTTTTCTTCCTGACGAATGTGC
Ifnb1	GGAAAGATTGACGTGGGAGATGTC	CAGTTTTGGAAGTTTCTGGTAAGTCTTCG
r18s	CTTAGAGGGACAAGTGGCG	ACGCTGAGCCAGTCAGTGTA
