# Supplementary material for: Coagulation proteases modulate nucleic acid uptake and cGAS-STING-IFN induction in the tumor microenvironment
Source: JCI Insight. 2025 Jul 22;10(17):e190311. doi: 10.1172/jci.insight.190311 (PMC12487691; doi:10.1172/jci.insight.190311)
Supplement: Unedited blot and gel images [file jciinsight-10-190311-s226.pdf]

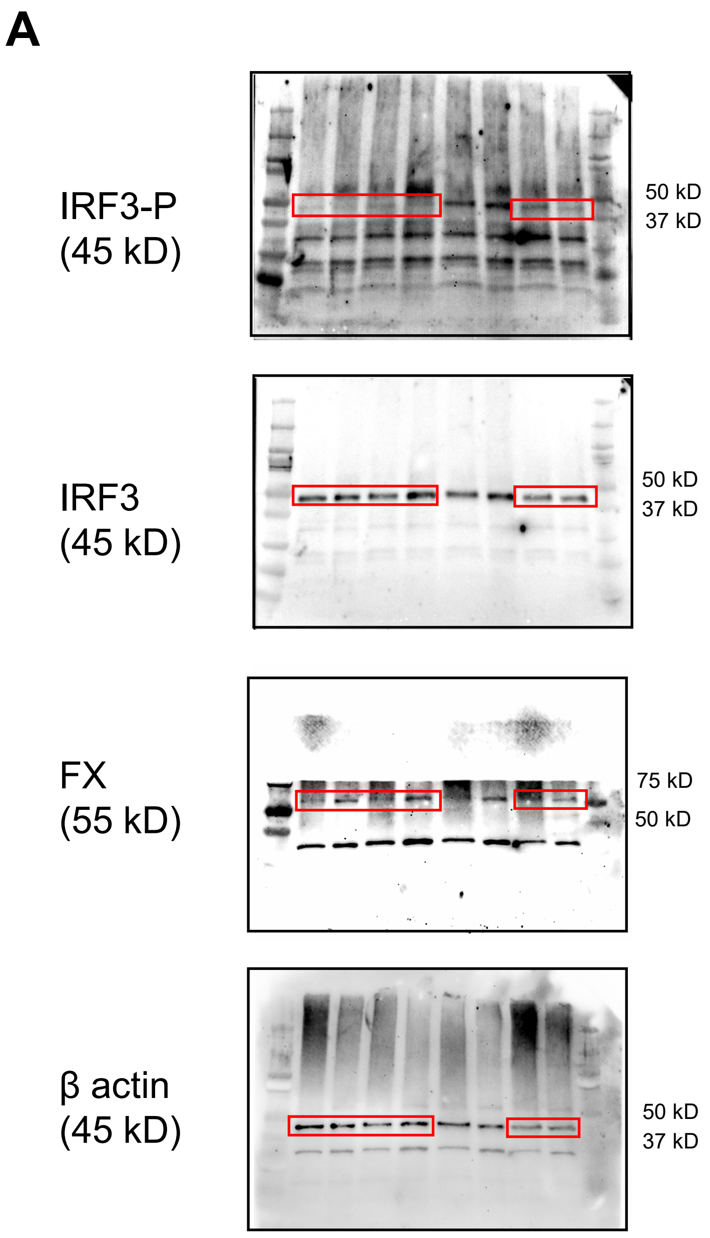

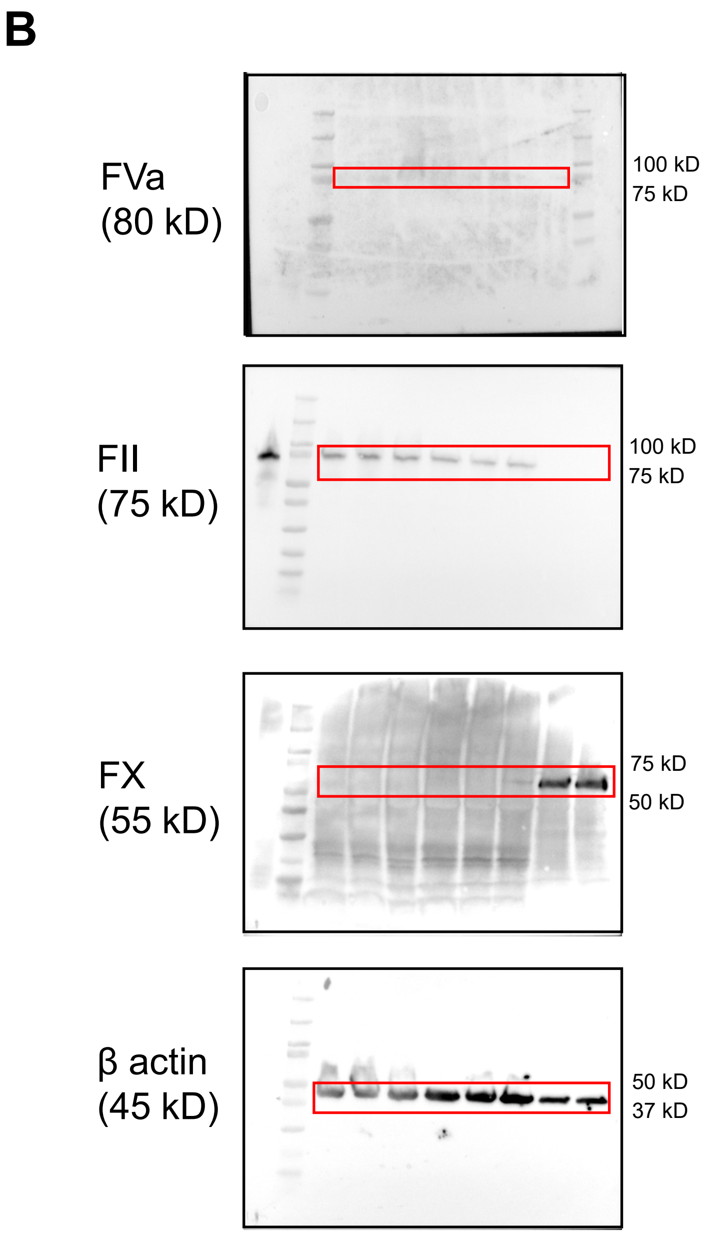

A) Full unedited gel for Figure 3C.
Representative blots were marked with

B) Full unedited gel for Figure 4B.
Representative blots were marked with
